# On the Question of Uncatalyzed CO Insertion into a Hydrazone Double Bond: A Comparative Study Using Different CO Sources and Substrates

**DOI:** 10.1021/acs.joc.4c00936

**Published:** 2024-06-18

**Authors:** Dongning Liu, Nicola Bauer, Wen Lu, Xiaoxiao Yang, Binghe Wang

**Affiliations:** Department of Chemistry and Center for Diagnostics and Therapeutics, Georgia State University, Atlanta, Georgia 30303, United States

## Abstract

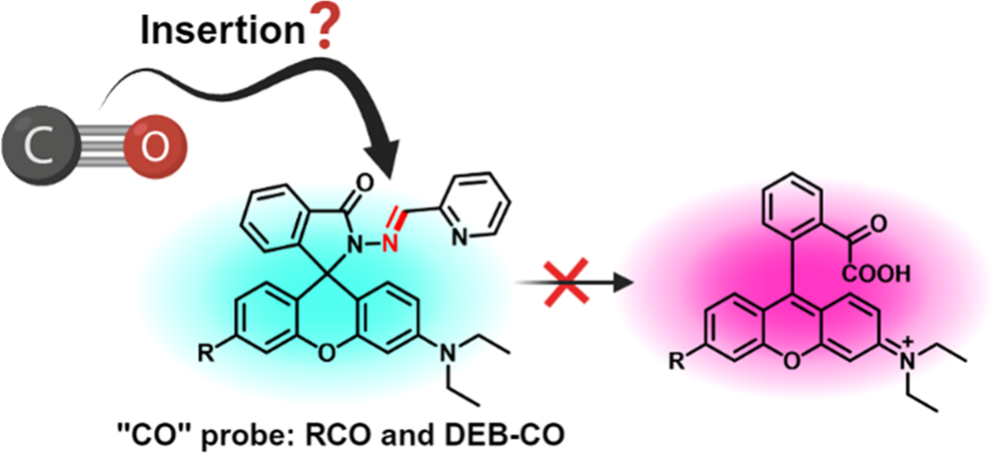

Because of endogenous signaling roles of carbon monoxide (CO) and its demonstrated pharmacological effects, there has been extensive interests in developing fluorescent CO probes. Palladium-mediated CO insertion has been successfully used for such applications. However, recent years have seen many publications of using uncatalyzed CO insertion into a hydrazone double bond as a way to sense CO. Such chemistry has no precedents otherwise. Further, the rigor of the CO-sensing work was largely based on using ruthenium–carbonyl complexes such as CORM-3 as CO surrogates, which have been reported to have extensive chemical reactivity and to release largely CO_2_ instead of CO unless in the presence of a strong nucleophile such as dithionite. For all of these, it is important to reassess the feasibility of such a CO-insertion reaction. By studying two of the reported “CO probes” using CO gas, this study finds no evidence of CO insertion into a hydrazone double bond. Further, the chemical reaction between CO gas and a series of eight hydrazone compounds was conducted, leading to the same conclusion. Such findings are consistent with the state-of-the-art knowledge of carbonylation chemistry and do not support uncatalyzed CO insertion as a mechanism for developing fluorescent CO probes.

## Introduction

**Scheme 1:**
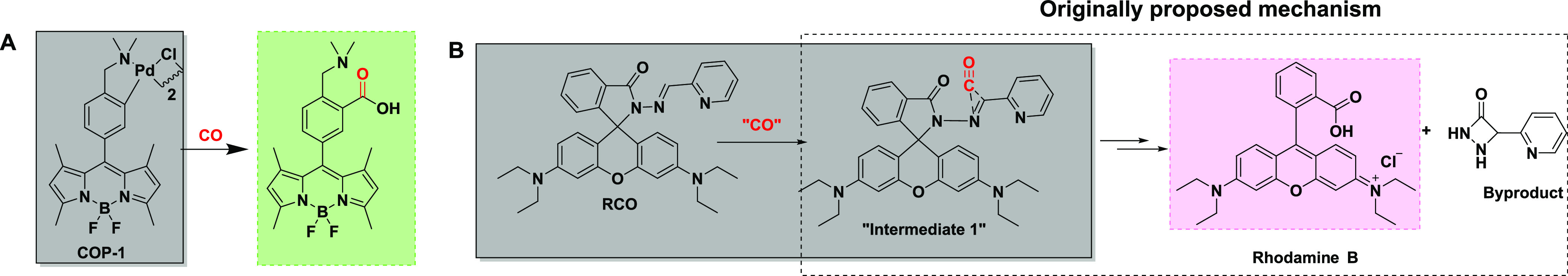
(A) The CO-Sensing Mechanism for COP-1; (B) The Originally Proposed CO-Sensing Mechanism for RCO through an Unprecedented “CO-Insertion” Reaction into a Hydrazone Double Bond

Carbonylation occupies a very special place in organic synthesis and represents some of the most useful reactions to enable access to a wide range of carbonyl containing intermediates.^1,2^ All known carbonylation reactions require catalysis by a transition-metal complex, including various complexes of ruthenium, rhodium,^3^ iron,^4^ palladium,^5^ cobalt,^6^ and copper.^7^ Interestingly, palladium-mediated carbonylation has been very cleverly used by Chang and colleagues for developing fluorescent probes for detecting carbon monoxide (CO) in biological settings (Scheme 1A).^8^ One can safely say that this publication by Michel, Lippert, and Chang started a wave of work on designing new small-molecule fluorescent probes for CO with various designs,^9^ including the use of Pd-catalyzed carbonylation and Pd-mediated deallylation for fluorophore activation in CO detection (Table S2).^10,11^ We are very interested in such work because of our interest in carbonylation chemistry,^12^ CO detection,^13^ CO biology,^14,15^ and CO donors as potential therapeutic agents.^16,17^ Our interest in CO largely stems from the fact that CO is an endogenously produced molecule in mammals with demonstrated pharmacological effects.^18−22^

Among the innovative use of chemistry in designing new CO probes, one recent development caught our attention: uncatalyzed carbonylation through a proposed CO insertion into a hydrazone double bond followed by hydrolytic generation of a fluorophore, rhodamine B (Scheme 1B).^23^ The proposed CO-insertion study provided little structural evidence of the resulting product formed except for a mass spectrometric peak that was identified by showing a narrow mass range of less than 2 Da. Since the initial report of such a CO-insertion reaction in 2019, this paper has been cited about 90 times. Further, the same chemistry has since been used in other designs of fluorescent CO probes including those published in 2022^24^ and 2023^25,26^ with extensive implications including CO imaging and detection in animal models, and the “accurate diagnosis of non-alcoholic fatty liver disease” in animal models.^25^ With such a broad impact in chemistry and biology of this type of chemistry and the continuous publication of new results using such chemistry, we are interested in understanding the feasibility and scope of the proposed CO-insertion reaction. In doing so, we focus on the work described in the original publication since that is the origin of this type of design. Along this line, we planned two types of studies: (1) studying the effect of CO on the original fluorescent probe (RCO) and an additional one (DEB-CO)^24^ by using CO from different sources and (2) examining the proposed CO-insertion reaction using a range of hydrazone substrates. The first type of study is necessary because the original study and subsequent publications used one ruthenium–carbonyl complex, CORM-3, as the primary CO source to establish the rigor of the study. With the known chemical reactivity of these Ru-carbonyl complexes^27−31^ and the stunning nature of the proposed uncatalyzed CO-insertion reaction, we feel additional experiments with a pure CO source are needed to confirm whether the fluorescent probe indeed detects CO via CO insertion. The second type of study will examine the intrinsic chemical feasibility of uncatalyzed CO insertion into a hydrazone double bond. Below, we describe our studies.

## Results and Discussion

### Effect of CO Gas on RCO and DEB-CO

For this reassessment work, we start with experimental work using RCO because of its role in initiating this line of work. We synthesized RCO using two independent literature procedures and confirmed its identity using NMR and MS (Figures S4–S10).^23,24^

The most straightforward way for us to observe CO’s reaction with RCO is through monitoring structural changes by an appropriate method such as NMR. To probe this, pure CO gas was directly bubbled into the MeOH-*d*_4_ solution of RCO at 1 mM for 5 min (at 20 °C, the solubility of CO in methanol is around 7 mM^32^) and then incubated at 37 °C for 30 min under seal. NMR spectra showed no change before and after incubation with CO, giving no evidence of CO insertion into the hydrazone double bond (Figures S32 and S33).

We conducted similar studies with another CO probe (DEB-CO), which was developed based on the same mechanism.^24^ Briefly, we synthesized DEB-CO and established its identity by NMR and MS (Figures S12–S15). We did similar ^1^H NMR studies for DEB-CO in DMSO-*d*_6_ due to its poor solubility in methanol. Again, we saw no changes in NMR spectra before and after incubation with CO (Figures S34 and S35).

Overall, from an organic chemistry point of view, the above studies with RCO and DEB-CO indicate no reaction between CO and these two “CO probes.” Next, we are interested in examining whether such results are consistent with fluorescent studies of these two probes since the intention of the original publications was for these compounds to achieve fluorescent detection of CO.

### Fluorescence Changes of RCO and DEB-CO Upon Addition of a CO Surrogate, CORM-3, and CO Gas

In examining the effect of CO on the fluorescent intensity of RCO and DEB-CO, we conducted three lines of work: (1) reproduction of the original experimental work by using CORM-3 as the CO source, (2) quantitative analyses of what the fluorescent results might mean, and (3) assessment of the probe fluorescent response to gaseous CO.

### Using CORM-3 as the Source of CO

**Figure 1:**
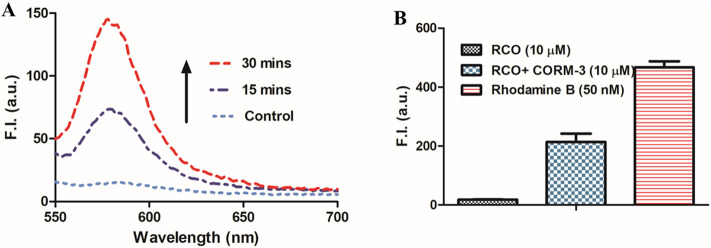
Effects of CORM-3 on the fluorescence of RCO solution (10 μM, in 10% DMSO, 90% PBS solution). (A) Fluorescence spectra after CORM-3 (10 μM) addition to the RCO solution (RCO only was used as a control). (B) Comparison of the fluorescence intensity of the CORM-3 (10 μM)-RCO solution (10 μM), rhodamine B (50 nM), and RCO probe only (10 M) (*n* = 3, mean ± SD, λ_ex_ = 530 nm, λ_em_ = 580 nm bandwidth = 5 nm).

Over the last 20 years, several metal/borane-carbonyl complexes have been widely used as CO surrogates for studying CO biology. They are named as CO-releasing molecules (abbreviated as CORMs) with CORM-2, CORM-3, CORM-401, and CORM-A1 being commercially available and thus most widely used. In the original study of using RCO to detect CO, CORM-3 (a ruthenium–carbonyl complex) was used as the source of CO for assessment. Though it is named as a CO-releasing molecule, it mostly releases CO_2_ instead of CO in aqueous buffer unless there is an added nucleophile, such as sodium dithionite.^29,33−35^ Furthermore, CORM-3 has extensive chemical reactivity as a reducing agent, catalase mimic, and an electrophile.^27,28,31,36^ In the original report, RCO (10 μM) was incubated with 1 equiv of CORM-3 in 10% dimethyl sulfoxide (DMSO) and 90% phosphate base saline (PBS) for 30 min, leading to an obvious fluorescent intensity increase. We conducted the same experiments and observed the same results, indicating reproducibility of the original experimental observation. Specifically, Figure 1A shows time-dependent fluorescent intensity increases at 580 nm (λ_ex_ = 530 nm). Then, there is the question of how to explain the fluorescent results in the context of the NMR studies described in the preceding paragraph. In the next section, we conduct a quantitative assessment of the fluorescence intensity changes observed to shed light on this issue.

### What the Fluorescence Increases Mean in a Quantitative Sense

There are good reasons that fluorescence has been widely used for the sensitive detection of analytes because it is highly sensitive. In contrast, NMR does not allow for reliable detection below a fraction of 1%. Could the inconsistency between the NMR results and fluorescence results be due to this sensitivity difference? For this, we measured the fluorescence of rhodamine B, the proposed product after RCO’s reaction with CO (Scheme 1B). As shown in Figure 1B, the fluorescence intensity of rhodamine B (positive control) at 50 nM is far higher than that of the reaction mixture between RCO and CORM-3 at 10 μM each. Such results mean that if rhodamine B was indeed formed in the reaction as proposed, the yield would be less than 0.5% based on fluorescence intensity (Figure S4 shows standard curves for rhodamine B in different solvents). Furthermore, there was no characterization of the fluorescent species in the original publication. Therefore, the nature of the reaction is unknown.

Considering all of the results thus far, the highly improbable nature of uncatalyzed CO insertion, the known complex nature of CORM-3 chemistry, the lack of structural characterization of the product between RCO and CORM-3, and the meniscal amount of fluorescent product, we can say with a high degree of certainty that there is no evidence to support CO insertion into hydrazone double bond as a way to sense CO by RCO. Further, it has been shown before that sensing a CORM does not equate to sensing CO,^37^ and CORM-3 does not release a meaningful amount of CO unless there is a strong nucleophile.^34,38^ We did not expend extra efforts to examine the mechanistic details because of the known complexity of the Ru-carbonyl complexes in terms of its chemical reactions including redox properties, catalase-like activities, and reactivity with nucleophiles. However, it should be noted that there are reports of enhanced fluorescence intensity of fluorophores through complexation with Ru(II).^39,40^ Further, a Ru(II) complexation with hydrazone has been reported.^41^ The fluorescence changes described in the original papers are likely due to Ru-mediated events, which further support the theme of this study: uncatalyzed CO insertion does not happen. The scope of this paper remains focused on examining whether CO insertion into hydrazone double bond was the reason for the reported fluorescent turn-on and do not wish to make this a ruthenium chemistry project, which would go beyond the scope of this study.

**Figure 2:**
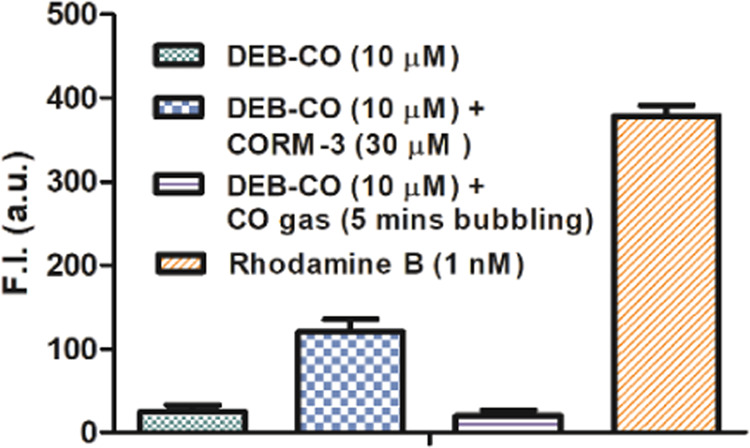
Comparison of the fluorescence intensity of the DEB-CO solutions (10 μM, in 5% DMSO, 95% HEPES solution) incubated with CORM-3 (30 μM) for 30 min at 37 °C; DEB-CO were probed with CO gas by bubbling CO gas for 5 min, then incubating at 37 °C for 30 min (DEB-CO only and 1 nM rhodamine B were used as a control, *n* = 3, mean ± SD, λ_ex_ = 580 nm, λ_em_ = 630 nm, bandwidth = 10 nm).

With the demonstration of the minuscule level of fluorescent intensity changes from RCO and CORM-3, we sought to examine a second case (DEB-CO) briefly just to see whether we would have similar experimental observations. When CORM-3 (3 equiv) was added to 10 μM DEB-CO solution in HEPES buffer and incubated at 37 °C, the solution showed a weak fluorescence intensity increase after 30 min of incubation. The fluorescence intensity was lower than that of 1 nM rhodamine B (the original paper used rhodamine B as their quantum yield standard, although the proposed end product is only a derivative of rhodamine B). When we compared rhodamine B to the reaction mixture of DEB-CO (10 μM) and CORM-3 (30 μM), the fluorescent intensity indicated the generation of a negligible percentage (<0.01%) of fluorophoric compound from the parent DEB-CO (Figure 2). The results from DEB-CO were less than that of the original publication and the identity of the weakly fluorescent species from DEB-CO after incubation with CORM-3 is not clear.

With all of the findings that are inconsistent with uncatalyzed CO insertion into a hydrazone double bond to a meaningful degree, we sought to conduct additional confirmation experiments using pure CO gas.

### Experiments with CO Gas

**Figure 3:**
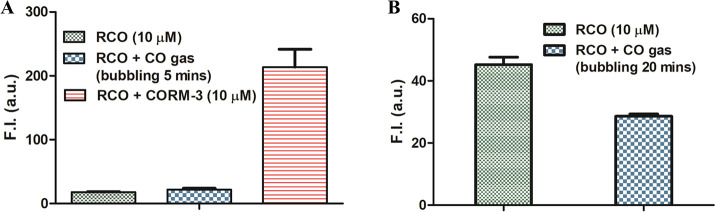
Effects of CORM-3 and CO gas on the fluorescence of RCO solution (10 μM, in 10% DMSO, 90% PBS solution). (A) RCO solutions were probed with CO gas by bubbling CO gas for 5 min, then incubating at 37 °C for 30 min. (B) RCO solutions were probed with CO gas by bubbling CO gas for 20 min, then incubating at 37 °C for 18 h (probe only was used as control) (*n* = 3, mean ± SD, λ_ex_ = 530 nm, λ_em_ = 580 nm bandwidth = 5 nm).

To lay any claim to a new CO-insertion reaction, it must be able to undergo the same reaction with CO gas. Thus, pure CO gas was used to assess the true CO-sensing ability of RCO and DEB-CO. Briefly, for RCO, CO gas was bubbled into a RCO solution (10% DMSO, 90% PBS solution) for 5 min. As shown in Figure 3A, there was no meaningful difference in fluorescence intensity between the CO group and the control group (RCO 10 μM), after 30 min of incubation. In comparison, 1 equiv CORM-3 (10 μM) was able to cause a small increase in the fluorescence of RCO within 30 min. These results show that RCO only responded to CORM-3, but not CO. It should be noted that in the original publication, experiments were also conducted using CO gas, which was said to increase the fluorescence of RCO by an estimated 6-fold after 12 h of incubation (Figure S52). We conducted similar experiments using 1 mM CO in solution. Briefly, RCO solution (10 μM) was sealed in a vial. Then pure CO gas was bubbled into the RCO solution for 20 min followed by incubation at 37 °C for 18 h. No fluorescence intensity change was observed (Figure 3B). We have no explanation of the different experimental observations in using CO gas.

For DEB-CO, we conducted similar studies by using pure CO gas. Briefly, pure CO gas was bubbled into a DEB-CO solution (5% DMSO, 95% HEPES solution) for 5 min followed by incubation at 37 °C for 30 min. We saw no indication that DEB-CO possessed meaningful ability to sense CO gas (Figure 2, column 3). Such results indicate that DEB-CO did not respond to or react with CO and is not a probe for CO. Comparative studies were carried out using CORM-3, leading to marginal fluorescence intensity increases. Importantly, such results are different from what was reported in the original publication.^24^ Again, we have no explanation of the different experimental observations.

Overall, our results do not support an uncatalyzed CO-insertion reaction to lead to fluorescence response of RCO and DEB-CO to CO in a meaningful fashion. To our knowledge, there is no known chemical pathway for the originally proposed CO insertion into a hydrazone bond without metal catalysis. The establishment of such a novel uncatalyzed CO-insertion reaction will require much more rigorous evidence than presented in the original publication.

### Assessing Uncatalyzed CO Insertion into Additional Hydrazone Analogs: No Evidence for CO Insertion

Though experiments using RCO and DEB-CO with CORM-3 and CO did not lead to any evidence of the proposed uncatalyzed CO insertion into hydrazone, for the sake of thoroughness, we conducted additional experiments to examine the chemical feasibility of the proposed CO-insertion reactions using various hydrazone analogs. For this, eight hydrazone compounds (Table 1) were synthesized by following published procedures (see Supporting Information, SI for details).^42^ The identities of these compounds were confirmed by NMR (both ^1^H and ^13^C) and MS. Interestingly, the NMR spectra of compounds **5**-**8** (Figures S24–S31) showed two sets of peaks of the methylene group. The stereoisomerism issue and associated NMR characterization work involving such structures have been comprehensively studied and addressed by Munir, Zia-ur-Rehmen, and coauthors in a very interesting 2021 publication.^43^ Herein, we stay focused on the CO-insertion question.

**Table 1:**
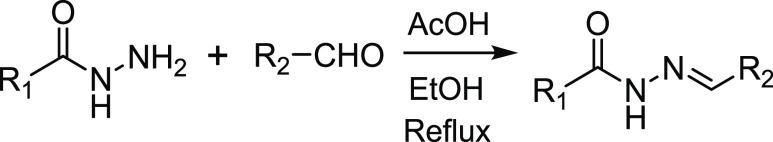

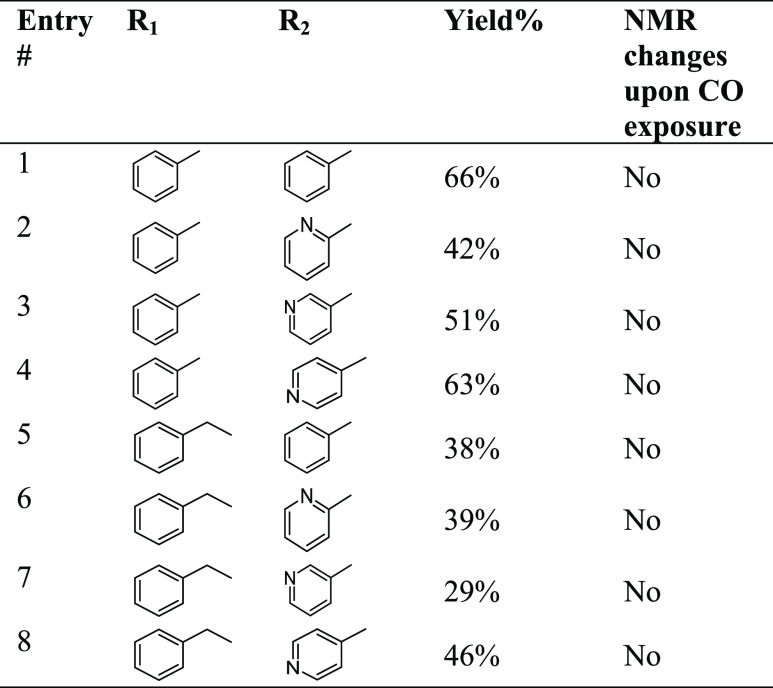
Summary of Hydrazone Compounds

To assess the reactivity between a hydrazone compound and CO gas, NMR experiments with these eight hydrazone compounds were conducted. Specifically, pure CO gas was bubbled into a 1 mM MeOH-*d*_4_ solution of the hydrazone compound for 5 min followed by incubation of the sealed tube at 37 °C for 30 min. ^1^H NMR spectra were collected before and after CO incubation. We observed no changes in the NMR spectra as a result of exposure to CO (Figures S35–S50). Figures S35 and S36 show one representative set of the spectra. It is concluded that the NMR evidence presented does not support any reaction between these hydrazone compounds and CO gas under the experimental conditions.

In conclusion, a new uncatalyzed CO-insertion reaction into hydrazone double bond was proposed in the context of designing new fluorescent probes for CO by a 2019 publication. The original paper has drawn widespread attention, has been cited 90 times, has led to subsequent publications using the same type of design, and has been used in high-impact applications such as “accurate diagnosis of non-alcoholic fatty liver disease.” Given the broad impact of this astonishingly improbable reaction, we have reassessed the ability for CO to insert into a hydrazone double bond. This was done by assessing the ability for the originally proposed CO probes to sense pure CO and by conducting CO-insertion reactions using a series of 8 hydrazone analogs. None of our results support the feasibility of uncatalyzed CO insertion into a hydrazone double bond as a way to sense CO. Just as important to demonstrate the improbable nature of the originally proposed CO-insertion reaction, the problems associated with ruthenium-based CORMs (and some other CORMs) as CO surrogates should also be emphasized. It is important to note that four independent laboratories have shown that these ruthenium–carbonyl complexes do NOT produce a meaningful amount of CO in buffer and cell culture media unless there is an added nucleophile, sodium dithionite.^14,33,34,44,45^ The case study described here is another example that these ruthenium-based CORMs (CORM-2 and CORM-3) should not be used to study CO biology or CO chemistry and should not be used to further develop new CO probes.

## Data Availability

The data underlying this study are available in the published article and its Supporting Information.

## Supporting Information Available

Additional details on the synthetic procedures, experimental work, spectroscopic data and spectra, figures, and tables with hydrazone compounds synthesized as well comparative data of published CO probes (PDF)
