# Supplementary material for: On the Question of Uncatalyzed CO Insertion into a Hydrazone Double Bond: A Comparative Study Using Different CO Sources and Substrates
Source: J Org Chem. 2024 Jun 18;89(13):9551–6. doi: 10.1021/acs.joc.4c00936 (PMC11232009; doi:10.1021/acs.joc.4c00936)
Supplement: Supplementary file 1 — jo4c00936_si_001.pdf [file jo4c00936_si_001.pdf]

Supporting Information

On the question of uncatalyzed CO insertion into a hydrazone double bond: A comparative study using different CO sources and substrates

Dongning Liu, Nicola Bauer, Wen Lu, Xiaoxiao Yang and Binghe Wang*

Department of Chemistry and Center for Diagnostics and Therapeutics, Georgia State University, Atlanta, Georgia 30303, USA

Table of contents

Supporting Figures S2
Experimental details S4
NMR spectra and MS data S11
Figures from original publications S40

Supplemental Figures

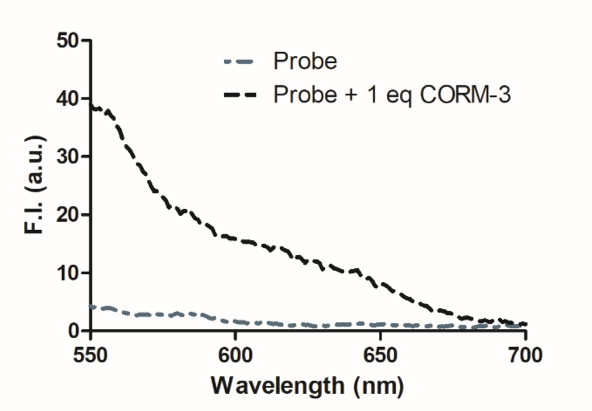

Figure S1. The fluorescence of RCO (10 μM) response to CORM-3 (10 μM). RCO only was used as a control. (λ_{ex} = 530 nm, bandwidth = 10 nm).

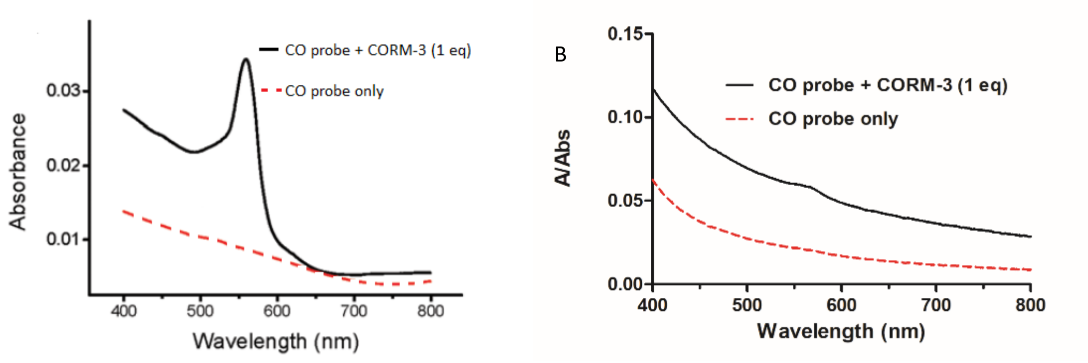

Figure S2. Effects of CORM-3 on the absorbance of RCO solution. A. Original absorption spectra of 10 μM RCO in PBS solution after the addition of CORM-3 (10 μM) and 30 mins incubation at 37°C. Taken from "Chem. Commun., 2019, 55, 9444" with modification. B. The effects on the absorbance of RCO solution (10 μM, in 10% DMSO, 90% PBS solution) after addition of CORM-3 (1 eq) and 30 mins incubation at 37°C. (RCO probe was used as a control).

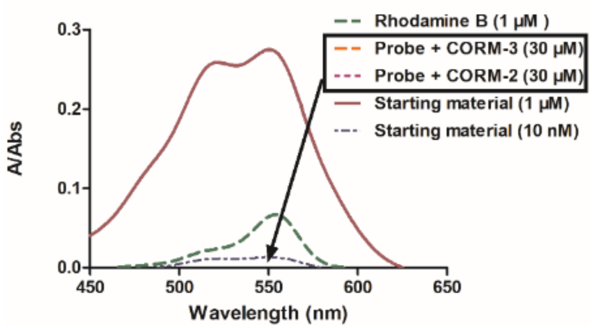

Figure S3 Comparison of UV-Vis absorption of DEB-CO (10 μM, in 5% DMSO, 95% HEPES solution) incubated with CORM-2/CORM-3 and rhodamine B (1 μM), and starting material (1 μM and 10 nM). (Probe only and 1 nM Rhodamine B were used as a control, n = 3, mean ± SD, λ_{ex} = 580 nm, λ_{em} = 630 nm bandwidth = 10 nm).

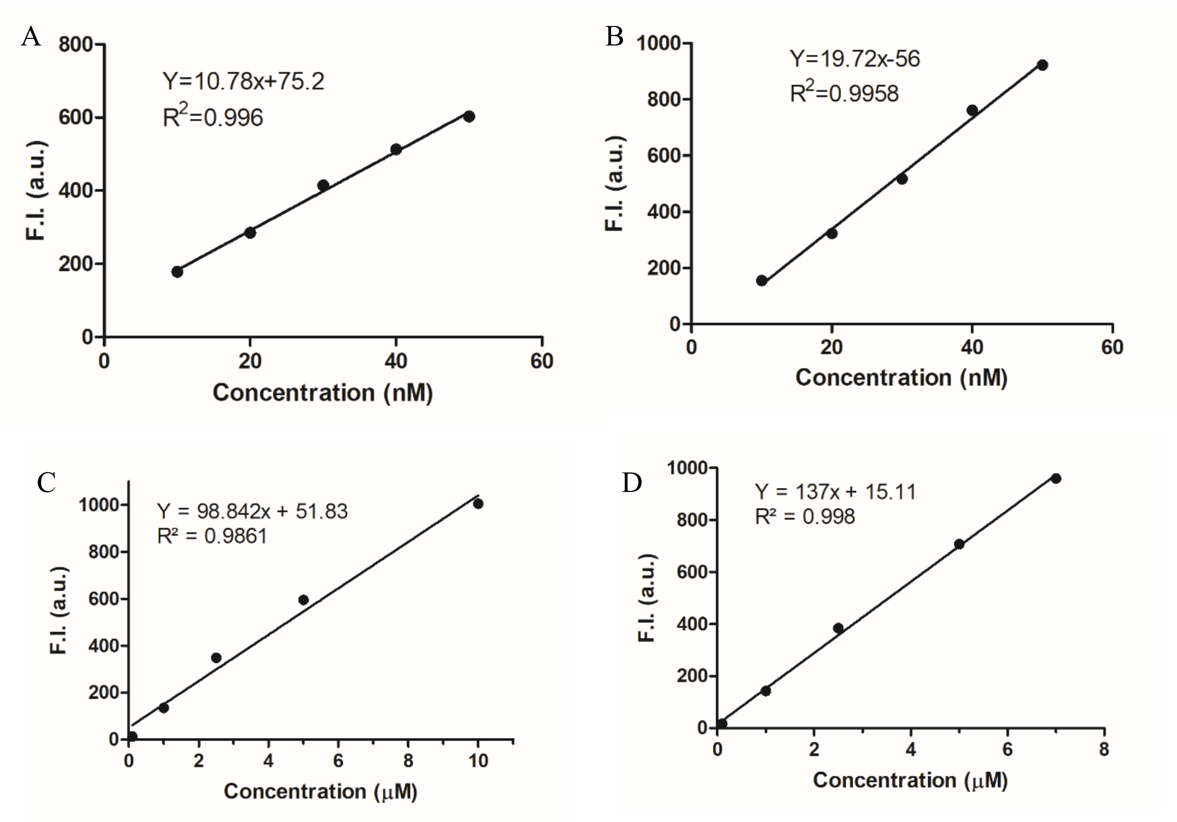

Figure S4. Fluorescence standard curves of rhodamine B in different solvents. A. Standard curve of rhodamine B in PBS solution in the nanomolar range (λ_{ex} = 530 nm, λ_{em}= 574 nm bandwidth = 5 nm) B. Standard curve of rhodamine B in 90% PBS and 10% DMSO solution in the nanomolar range (λ_{ex} = 530 nm, λ_{em}= 574 nm, bandwidth = 5 nm) C. Standard curve of rhodamine B in PBS solution in the micromolar range (λ_{ex} = 530 nm, λ_{em}= 574 nm Ex bandwidth = 3 nm Em bandwidth= 1.5 nm) D. Standard curve of rhodamine B in in 90% PBS and 10% DMSO solution in the micromolar range (λ_{ex} = 530 nm, λ_{em}= 574 nm Ex bandwidth = 3 nm Em bandwidth= 1.5 nm).

Experimental details

General Materials and Methods. Chemical reagents were purchased from Sigma-Aldrich (Saint Louis, Missouri, USA) and/or Oakwood (Estill, South Carolina, USA) and/or AA Blocks(San Diego, Carlifornia, USA). Solvents were purchased from Fisher Scientific (Pittsburgh, Pennsylvania, USA); dry solvents were prepared using a Vigor Tech purification system (Houston, Texas, USA). Certificated pure CO calibration gas was purchased from GASCO (Oldsmar, Florida, USA). UV-Vis absorption spectra were obtained by using a Shimadzu PharmaSpec UV-1700 UV-Visible spectrophotometer (Kyoto, Japan). Fluorescence spectra were recorded on a Shimadzu RF5301PC fluorometer (Kyoto, Japan). ^{1}H-NMR (400 MHz) and ^{13}C-NMR (101 MHz) were acquired with Bruker AV-400 MHz Ultra Shield NMR.

Data for NMR are reported in terms of chemical shift (δ, ppm): Multiplicity (s = singlet, d =doublet, t = triplet, q = quartet, m = multiplet or unresolved, br = broad singlet, coupling constant(s) in Hz)

Synthesis of RCO

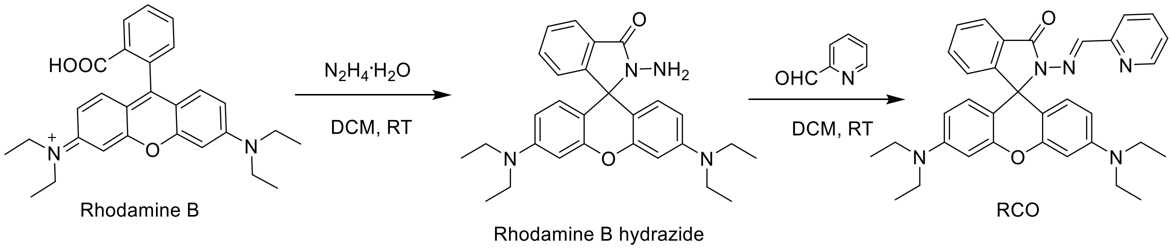

Scheme S1. The synthesis scheme of RCO.

RCO was synthesized by following a literature procedure.[1] Brifely, 90 mg rhodamine B (0.38 mmol), 0.25 mL hydrazine hydrate (3.8 mmol) and 190 mg BOP [benzotriazol-1-yloxytris(dimethylamino) phosphonium hexafluorophosphate] reagent (0.4 mmol) was dissolved in 10 mL DCM. The reaction mixture was stirred at room temperature for 6 h. Solvent was removed by rotary evaporator , followed by purification via chromatography (silica gel, DCM: MeOH = 20: 1). After drying, the pink solid product obtained was dirctly used for the next reaction.

In the second step, 90 mg (0.2 mmol) rhodamine B hydrazide was dissovled in 10 mL dry methnol. 0.1 mL picolinaldehyde (1.1 mmol) was added and the reaction mixture was stirred at room temperature overnight. Solvent was removed by rotary evaporator and the product was purified by column chromatography (silica gel, DCM :MeOH = 25:1) to yield a pink solid (30 mg, yield: 28%).

^{1}H NMR ($CDCl_3$): δ 8.46 (d, *J* = 4.9 Hz, 1H), 8.34 (s, 1H), 8.08 – 7.96 (m, 2H), 7.61 (s, 1H), 7.54 – 7.39 (m, 2H), 7.12 (dd, *J* = 13.3, 6.2 Hz, 2H), 6.55 (d, *J* = 8.8 Hz, 2H), 6.45 (d, *J* = 2.5 Hz, 2H), 6.23 (dd, *J* = 8.9, 2.5 Hz, 2H), 3.31 (q, *J* = 7.1 Hz, 8H), 1.14 (t, *J* = 7.0 Hz, 12H). ^{13}C {1H} NMR

($CDCl_3$): δ 165.8, 154.7, 153.0, 152.6, 149.1, 145.9,, 136.3, 133.9, 128.4, 128.0, 127.7, 123.8, 123.7, 120.8, 108.1, 98.4, 65.9, 44.4, 12.8. HRMS (ESI) m/z: $[M+H]^+$, 546.2872 (calcd. for $C_{34}H_{35}N_5O_2$, 546.2864).

In addition, we also synthesized RCO by using a different procedure.[2, 3] Brifely, 1.2 g rhodamine B (2.5 mmol) was dissolved in 30 mL EtOH; then 3.0 mL of hydrazine hydrate was added dropwise with vigorous stirring. The reaction mixture was heated under refluex for 2 h. The solution changed from dark purple to light orange and then became clear. Solvent was removed by rotary evaporator , followed by the slow addition of 1M HCl (50 mL) leading to a clear red solution. Then, 1M NaOH was used slowly to adjust the pH to 10. The resulting persipitate was filtered and washed three times by water. After drying, the pink solid product was obtained (0.98 g, yield 80%).

This rhodamine B hydrazide was directly used in the following step without purification. In the second step, 0.46 g rhodamine B hydrazide (1 mmol) was dissovled in 20 mL abosolute ethanol. 0.38 mL picolinaldehyde (4 mmol) was added and the reaction mixture was heated under reflux for 6 hours in an oil bath. The precipitate was filtered and washed three times with cold ethanol. The product was purified by column chromatography (silica gel, DCM : MeOH = 25:1) to yield a white solid (82 mg, yield: 15%). The compound prepared by this procedure is identical with the original one by comparing NMR.

Synthesis of DEB-CO

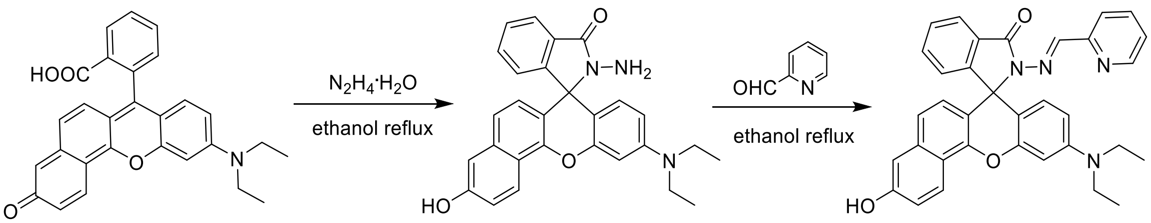

Scheme S2. The synthesis scheme of DEB-CO.

DEB-CO was synthesized by following a literature procedure.[2] Brifely, 0.44 g compound 1 (1 mmol) and 0.5 mL hydrazine hydrate were mixed in 15 mL ethanol. The reaction mixure was heated under reflux for 6 h in an oil bath. Then, the solvent was removed and the residue (DEB-HY) was purified by flash chromatography (silica gel, DCM: EA = 3:1) to yield a faint pink powder (115 mg, yield: 26%).

^{1}H NMR (400 MHz, DMSO-d_6): δ 8.36 (d, J = 9.1 Hz, 1H), 7.85 – 7.77 (m, 1H), 7.56 – 7.44 (m, 2H), 7.28 – 7.17 (m, 2H), 7.09 (d, J = 2.1 Hz, 1H), 7.04 – 6.93 (m, 1H), 6.63 (d, J = 1.7 Hz, 1H), 6.41 (dt, J = 12.3, 8.8 Hz, 3H), 4.35 (s, 2H), 3.34 (q, J = 6.9 Hz, 4H), 1.10 (t, J = 6.9 Hz, 6H). ^{13}C

{1H} NMR (100 MHz, DMSO-d_6): δ 166.0, 156.8, 152.8, 151.8, 148.7, 147.7, 135.7, 133.1, 129.8, 128.9, 128.0, 124.3, 124.0, 123.7, 122.7, 121.6, 118.8, 118.0, 110.5, 109.4, 108.8, 105.0, 97.9, 65.4, 44.0, 12.8. HRMS (ESI) m/z: $[M+H]^+$, 452.1981 (calcd. for $C_{28}H_{26}N_3O_3$, 452.1974).

In the second step, 115 mg DEB-HY (0.26 mmol) was dissolved in 10 mL abosolute ethonal, followed by the addition of 0.03 mL picolinaldehyde (0.3 mmol). Then the reaction mixture was heated under reflux for 6 h in an oil bath. The precipitate was filtered and washed by cold ethanol (10 mL*3). The product (DEB-CO) was obtained as a off-white powder with no further purification. Yield (40 mg, 27%) ^{1}H NMR (400 MHz, DMSO-d_6): δ 10.05 (s, 1H), 8.47 (d, J = 10.0 Hz, 2H), 8.40 (d, J = 4.2 Hz, 1H), 7.99 (d, J = 5.7 Hz, 1H), 7.73 (t, J = 7.8 Hz, 1H), 7.62 (dd, J = 12.2, 6.4 Hz, 3H), 7.31 – 7.21 (m, 3H), 7.15 – 7.05 (m, 2H), 6.74 (s, 1H), 6.60 (d, J = 8.7 Hz, 1H), 6.54 (d, J = 8.8 Hz, 1H), 6.42 (d, J = 7.6 Hz, 1H), 1.10 (t, J = 6.9 Hz, 6H). ^{13}C {1H} NMR (100 MHz, DMSO-d_6): δ164.3, 156.9, 153.1, 151.8, 151.5, 149.4, 148.8, 146.8, 136.6, 135.6, 127.7, 123.4, 119.2, 117.5, 109.8, 109.8, 104.3, 65.3, 12.52. HRMS (ESI) m/z: $[M+H]^+$, 541.2228 (calcd. for $C_{34}H_{29}N_4O_3$, 541.2240).

Synthesis of hydrazone compounds

Table S1. Summary of hydrazone compounds.

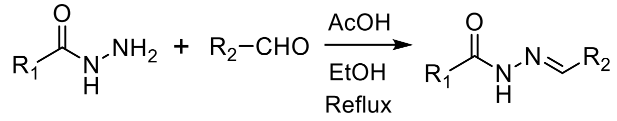

Entry #	R₁	R₂	Yield%	NMR changes upon CO exposure
1	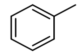	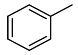	66%	No
2	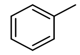	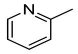	42%	No
3	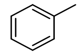	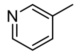	51%	No
4	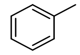	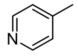	63%	No
5	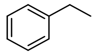	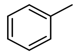	38%	No
6	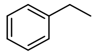	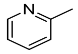	39%	No
7	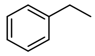	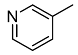	29%	No
8	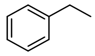	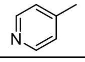	46%	No

General synthesis procedure: Hydrazone compounds have been synthesized by many groups,[4-6] herein we followed the same literature procedure for the synthesis of all these eight compounds.[7] In details, the hydrazide compound (2 mmol) was dissolved in 10 mL absolute ethanol, and then target aldehyde (2.4 mmol) was added along with a few drops (3-5 drops) of acetic acid. The reaction mixture was refluxed for 6 hours in an oil bath. After completion, the mixture was cooled down to room temperature, and the precipitate was collected, filtered, and recrystallized from ethanol to give the product.

As an example, for hydrazone compound (Entry 1), 270 mg (2 mmol) benzohydrazide was dissolved in 10 mL absolute ethanol. Then 255 μL (2.4 mmol) benzaldehyde was added along with a few drops (3-5 drops) of acetic acid. The reaction mixture was heated under reflux for 6 hours in an oil bath. Formation of white solid was observed. The mixture was cooled down to room temperature, and the precipitate was collected, filtered, and recrystallized from ethanol (20 mL) to give the product.

Hydrazone compound (entry 1): White crystal (296 mg, Yield: 66%). ^{1}H NMR (400 MHz, MeOD-*d*$_4$) δ 8.35 (s, 1H), 7.94 (d, *J* = 7.3 Hz, 2H), 7.84 (dd, *J* = 6.3, 2.8 Hz, 2H), 7.61 (t, *J* = 7.3 Hz, 1H), 7.53 (t, *J* = 7.5 Hz, 2H), 7.47 – 7.36 (m, 3H). ^{13}C {1H} NMR (101 MHz, MeOD-*d*$_4$) δ 167.2, 155.5, 150.7, 135.6, 134.2, 133.3, 131.6, 129.8, 129.8, 128.8, 128.8. HRMS (ESI) m/z: $[M+H]^+$, 225.1039 (calcd. for $C_{14}H_{13}N_2O$, 225.1028).

Hydrazone compound (entry 2): White solid (190 mg, Yield: 42%). ^{1}H NMR (400 MHz, MeOD-*d*$_4$) δ 8.83 (d, *J* = 4.4 Hz, 1H), 8.05 (dd, *J* = 11.0, 4.6 Hz, 1H), 8.00 (d, *J* = 7.3 Hz, 2H), 7.74 (d, *J* = 7.8 Hz, 1H), 7.69 – 7.49 (m, 5H). ^{13}C {1H} NMR (101 MHz, MeOD-*d*$_4$) δ 166.5, 153.6, 149.6, 141.2, 139.7, 133.8, 130.2, 128.6, 127.9, 126.1. HRMS (ESI) m/z: $[M+H]^+$, 226.0991(calcd. for $C_{13}H_{12}N_3O$, 226.0980).

Hydrazone compound (entry 3): Off-white solid (225 mg, Yield: 51%). ^{1}H NMR (400 MHz, MeOD-*d*$_4$) δ 8.90 (s, 1H), 8.57 (s, 1H), 8.39 (s, 2H), 7.95 (d, *J* = 7.3 Hz, 2H), 7.66 – 7.43 (m, 4H). ^{13}C {1H} NMR (101 MHz, MeOD-*d*$_4$) δ 167.3, 151.4, 149.9, 146.9, 136.1, 133.9, 133.5, 132.4, 129.8, 128.8, 125.5. HRMS (ESI) m/z: $[M+H]^+$, 226.0989 (calcd. for $C_{13}H_{12}N_3O$, 226.0980).

Hydrazone compound (entry 4): White solid (290 mg, Yield: 63%). ^{1}H NMR (400 MHz, MeOD-*d*$_4$) δ 8.59 (d, *J* = 5.2 Hz, 2H), 8.34 (s, 1H), 7.95 (d, *J* = 7.6 Hz, 2H), 7.83 (d, *J* = 5.2 Hz, 2H), 7.61 (d, *J* = 7.3 Hz, 1H), 7.53 (t, *J* = 7.5 Hz, 2H). ^{13}C {1H} NMR (101 MHz, MeOD-*d*$_4$) δ 167.3, 150.7, 147.1, 144.1, 133.7, 129.8, 128.9, 123.1. HRMS (ESI) m/z: $[M+H]^+$, 226.0977 (calcd. for $C_{13}H_{12}N_3O$, 226.0980).

Hydrazone compound (entry 5): White crystal (182 mg, Yield: 38%).^{1}H NMR (400 MHz, MeOD-*d*$_4$) δ 8.13 (s, 1H), 7.94 (s, 1H), 7.80 – 7.66 (m, 3H), 7.45 – 7.18 (m, 13H), 4.07 (s, 1H), 3.62 (s, 2H). ^{13}C {1H} NMR (101 MHz, MeOD-*d*$_4$) δ 176.0, 170.6, 149.9, 145.9, 136.3, 135.4, 131.6, 131.2, 130.5, 130.1, 129.9, 129.8, 129.7, 129.5, 128.8, 128.2, 128.1, 127.8, 42.3, 40.4. HRMS (ESI) m/z: $[M+H]^+$, 239.1191 (calcd. for $C_{15}H_{15}N_2O$, 239.1184).

Hydrazone compound (entry 6): Off-yellow solid (185 mg, Yield: 39%). ^{1}H NMR (400 MHz, MeOD-d_4) δ 8.57 – 8.47 (m, 2H), 8.27 – 8.12 (m, 2H), 8.06 – 7.95 (m, 1H), 7.87 (ddd, J = 15.4, 8.4, 1.3 Hz, 2H), 7.46 – 7.17 (m, 10H), 4.10 (s, 1H), 3.65 (s, 2H). ^{13}C {1H} NMR (101 MHz, MeOD-d_4) δ 176.0, 170.9, 154.4, 150.2, 150.1, 148.5, 144.6, 138.7, 138.6, 136.9, 136.1, 130.5, 130.1, 129.7, 129.5, 128.2, 127.8, 126.0, 125.7, 122.2, 121.7, 42.3, 40.3. HRMS (ESI) m/z: $[M+H]^+$, 240.1143 (calcd. for $C_{14}H_{14}N_3O$, 240.1137).

Hydrazone compound (entry 7): Off-yellow solid (139 mg, Yield: 29 %). ^{1}H NMR (400 MHz, MeOD-d_4) δ 8.81 (dd, J = 19.5, 1.6 Hz, 2H), 8.54 (dd, J = 4.8, 1.8 Hz, 2H), 8.29 (dt, J = 8.0, 1.8 Hz, 1H), 8.22 – 8.12 (m, 2H), 7.97 (s, 1H), 7.48 (ddd, J = 11.0, 8.0, 4.9 Hz, 2H), 7.39 – 7.13 (m, 8H), 4.08 (s, 1H), 3.64 (s, 2H). ^{13}C {1H} NMR (101 MHz, MeOD-d_4) δ 175.8, 170.8, 151.4, 151.0, 149.8, 149.1, 146.1, 142.1, 136.6, 136.1, 136.1, 135.6, 132.4, 132.2, 130.4, 130.1, 129.7, 129.5, 128.2, 127.8, 125.5, 125.5, 42.3, 40.5. HRMS (ESI) m/z: $[M+H]^+$, 240.1146 (calcd. for $C_{14}H_{14}N_3O$, 240.1137).

Hydrazone compound (entry 8): Off-yellow solid (220 mg, Yield: 46%)^{1}H NMR (400 MHz, MeOD-d_4) δ 8.62 – 8.51 (m, 4H), 8.13 (s, 1H), 7.92 (s, 1H), 7.76 (dd, J = 4.8, 1.4 Hz, 2H), 7.72 – 7.64 (m, 2H), 7.37 – 7.17 (m, 9H), 4.09 (s, 2H), 3.65 (s, 2H). ^{13}C {1H} NMR (101 MHz, MeOD-d_4) δ 175.9, 171.0, 150.7, 150.7, 146.3, 144.1, 143.9, 142.4, 136.4, 136.0, 130.5, 130.1, 129.9, 129.5, 128.2, 127.8, 123.0, 122.6, 42.4, 40.4. HRMS (ESI) m/z: $[M+H]^+$, 240.1127 (calcd. for $C_{14}H_{14}N_3O$, 240.1137).

Fluorescence of RCO and its response to CORM-3.

For Figure 1, RCO was prepared as a 500 μM stock solution in DMSO. As an example, 0.480 mg RCO was weighed using a microbalance followed by the addition 1.757 mL of DMSO to yield a 500 μM stock solution. Similarly, 0.271 mg CORM-3 was weighed, followed by the addition of 1.84 mL PBS solution (0.01 M) to yield a 500 μM stock solution. Subsequently, 900 μL PBS solution, 80 μL DMSO, and 20 μL of RCO stock solution were added into a cuvvette, resulting in a final RCO concentration of 10 μM. For experiments testing the RCO response to CORM-3, 880 μL PBS solution, 20 μL of RCO stock solution, 80 μL DMSO and 20 μL of CORM-3 stock solution were added into a cuvvette, resulting in a final DEB-CO and CORM-3 concentration of 10 μM. For the 50 nM rhodamine B solution, 1 mM stock solution was prepared in PBS. Then, 895 μL PBS, 100 μL DMSO and 5 μL of Rhodamine B stock solution, were mixed. These cuvvettes were sealed and incubated for 30 min at 37 °C, then fluorescence intensity of those samples reported at λ_{ex} = 530 nm, bandwidth = 5 nm.

UV-Vis of RCO and its response to CORM-3.

For Figure 2, the solutions described above were used for absorption measurements.

Fluorescence of RCO and its response to CO gas.

For testing RCO response to CO (Figure 3A), a 10 μM solution was prepared in a cuvvette using 20 μL of the RCO stock solution, 80 μL DMSO, 900 μL PBS solution. Pure CO gas was bubbled

via a syringe to the solution for 5 min in an open atmosphoere. After CO gas bubbling, the cuvvette was sealed. The cuvvette was then incubated at 37 ºC for 30 mins. Fluorescence intensity was measured at λ_{ex} = 530 nm, λ_{em} = 580 nm with bandwidth = 5 nm. For Figure 3B, the RCO solution was prepared in a sealed vial, and pure CO gas was bubbled via a syringe through the septum for 20 mins with an outlet to balance the pressure. The incubation time was 18 h. After incubation, fluorescence intensity was measured with same the settings of above.

Fluorescence of DEB-CO and its response to CORM or CO addition. For Figure 5, DEB-CO solutions were prepared as described in the original publication. DEB-CO was prepared as a 200 μM stock solution in DMSO. As an example, 0.261 mg DEB-CO was weighed using a microbalance and then 2.43 mL of DMSO was added to yield the stock solution. In a glass crimp seal vial, 2 mL of 50 mM HEPES buffer was added. Then, 100 μL of DEB-CO stock solution was added, resulting in a final DEB-CO concentration of 10 μM. For experiments testing the fluorescence of DEB-CO only, these vials were sealed and incubated on a shaker for 30 min at 37 ºC. For experiments testing the response to CORM-2 or CORM-3, 6.3 μL of CORM-2 or CORM-3 stock solution (10 mM in DMSO or water, respectively) was added to the original DEB-CO/HEPES solution before sealing, resulting in a final concentration of 30 μM CORM. The vial was then sealed and incubated on a shaker for 30 min at 37 ºC.

Recognizing that the orignal method actually produces 9.52 μM DEB-CO in the final solution, we also did the experiment where resulting DEB-CO solution was truly 10 μM, so DEB-CO and CORM-3 reactions were made with 1.9 mL of 50 mM HEPES, 100 μL of 200 μM DEB-CO stock in DMSO, and 6 μL of 10 mM CORM-3 stock. After incubating at 37 ºC for 30 min, we found minor differences with a F.I. of 158 ± 5.1, which is no significant difference with the previous results. For experiments testing CO response, the vial containing HEPES and DEB-CO was sealed. Pure CO gas was bubbled via syringe through the septum for 5 min. The vial was then incubated on a shaker for 30 min at 37 ºC. After incubation, fluorescence of either just DEB-CO, DEB-CO + CORM, or DEB-CO + CO gas was measured in a 1.5 mL cuvette. The spectrum was recorded at 37 ºC. Fluorescence intensity reported was taken at λ_{ex}= 580 nm, λ_{em}= 629 nm.

Fluorescence of rhodamine B for DEB-CO. Rhodamine B was prepared as a 10 mM stock solution in DMSO (ex: 4.344 mg in 900 μL DMSO). Solution was diluted to 1 mM rhodamine B in DMSO, then subsequent dilutions were done in HEPES buffer. Fluorescence was measured in a 1.5 mL cuvette and the spectrum was recorded at 37 ºC. Fluorescence intensity reported was taken at λ_{ex}= 556 nm, λ_{em}= 579 nm.

UV-Vis of rhodamine B, DEB-CO and DEB-CO response to CORM or CO addition. The solutions described above were removed from the cuvette and transferred to a UV-vis cuvette and tested the absorption spectrum.

NMR reaction of hydrazone compounds with CO gas. First, the hydrazone compounds were dissolved in MeOD-d_4 to prepare a 10 mM stock solution. The 50 μL stock solution was added into an NMR tube, then 450 μL MeOD-d_4 was added to the same NMR tube to get a 1 mM solution. Second, a long needle was attached to CO bags (The bags were filled with pure CO gas from the CO cylinder), and the needle was inserted into the bottom of the NMR tube, and the bag was squeezed with appropriate force, the stable formation of bubbles was observed. Finally, after bubbling CO gas for 5 mins, the NMR tube was sealed the with parafilm and incubated under 37 ^{o}C for 30 mins. The proton were collected again to compare with the previous spectrums.

MS data and NMR spectra

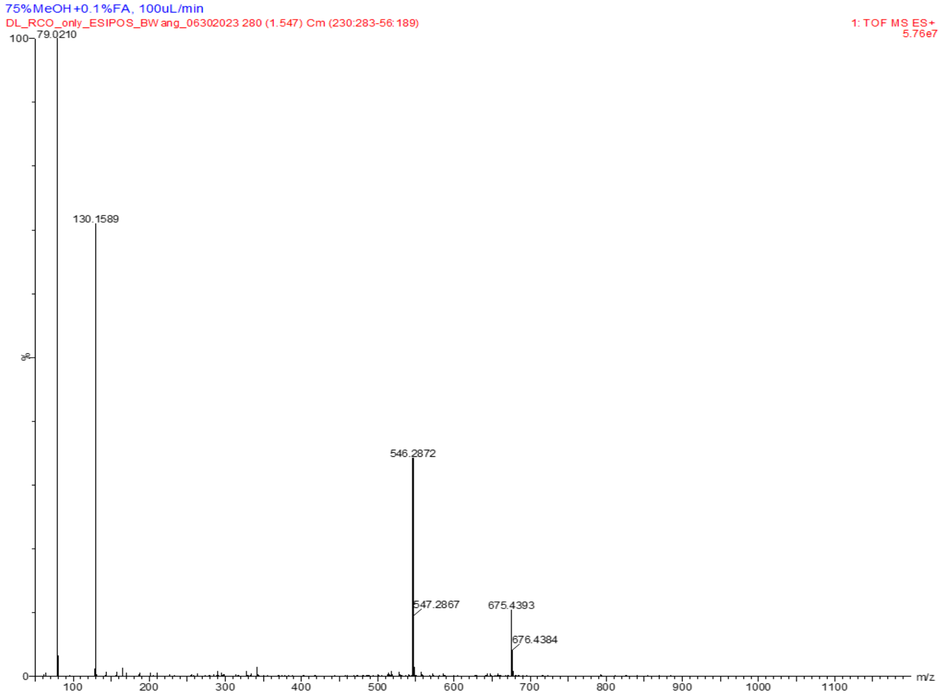

Figure S5. The mass spectrum of RCO. (Full spectrum)

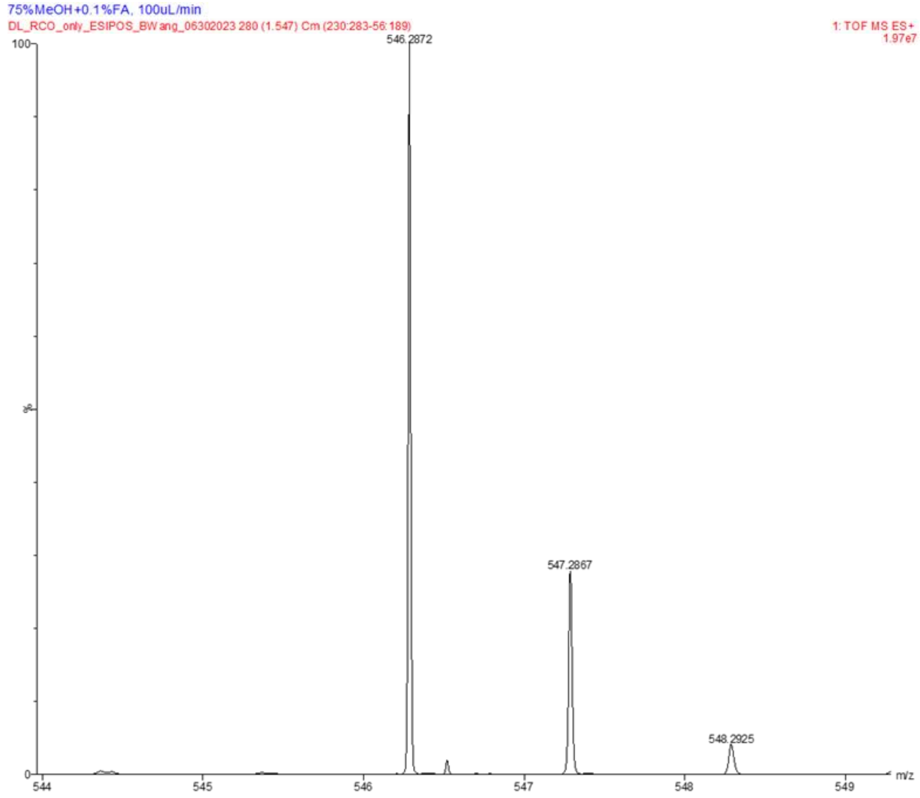

Figure S6. The mass spectrum of RCO. (Zoomed in a similar range with the original publication)

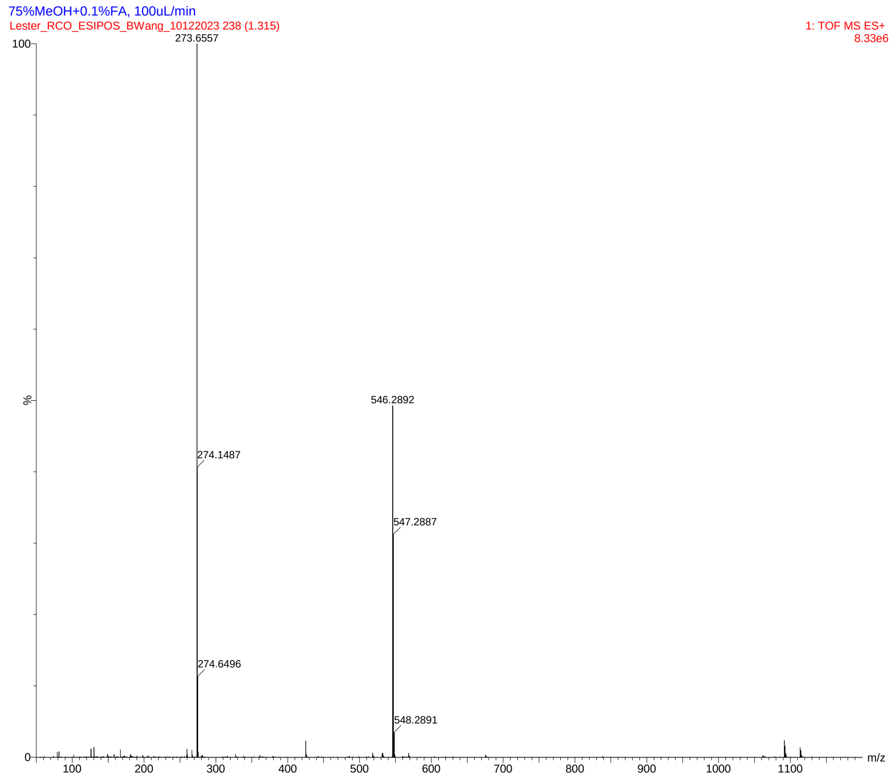

Figure S7. The mass spectrum of RCO without buffer.

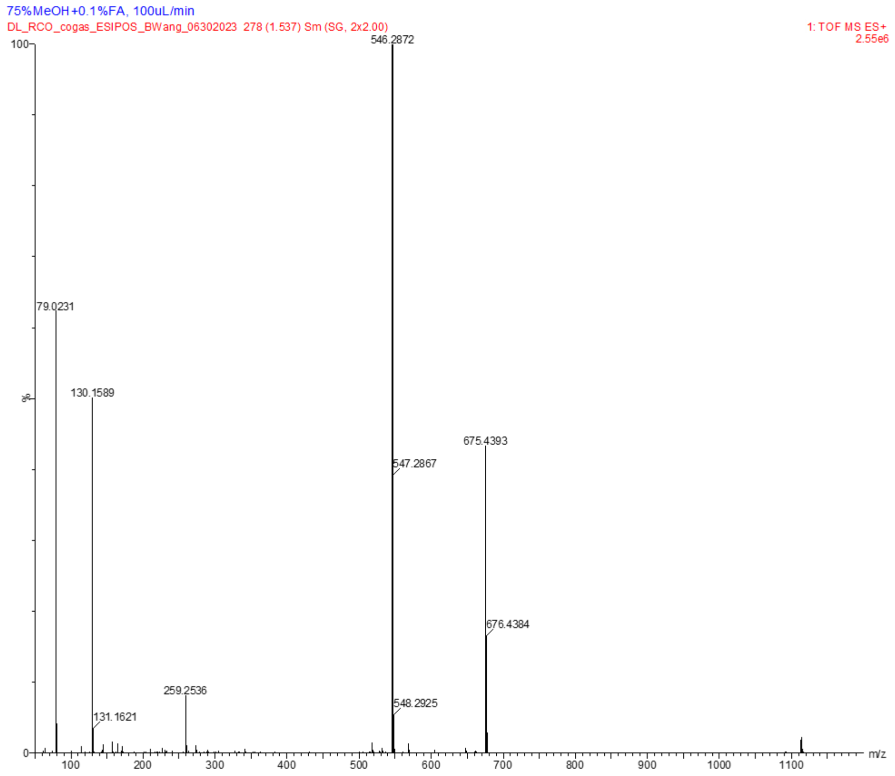

Figure S8. The mass spectrum of RCO and CO gas.

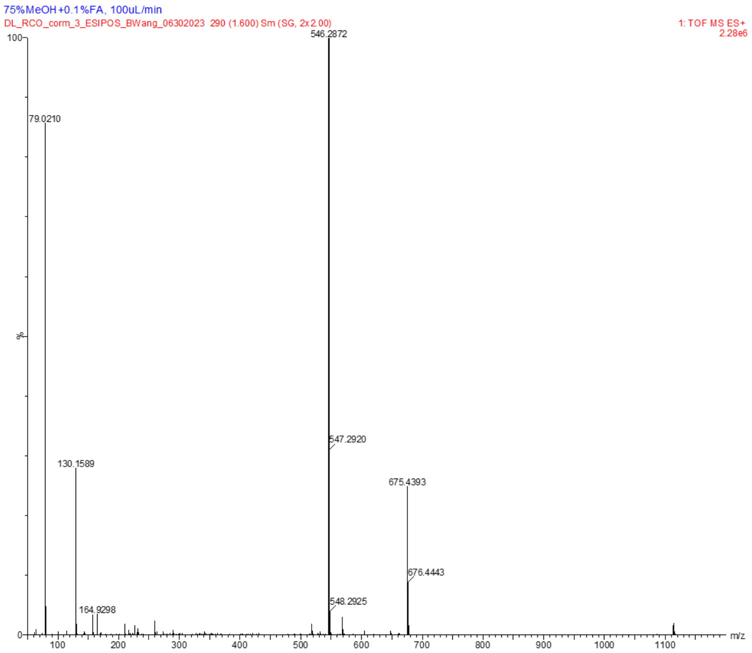

Figure S9. The mass spectrum of RCO and CORM-3 (1:1, 50 μM).

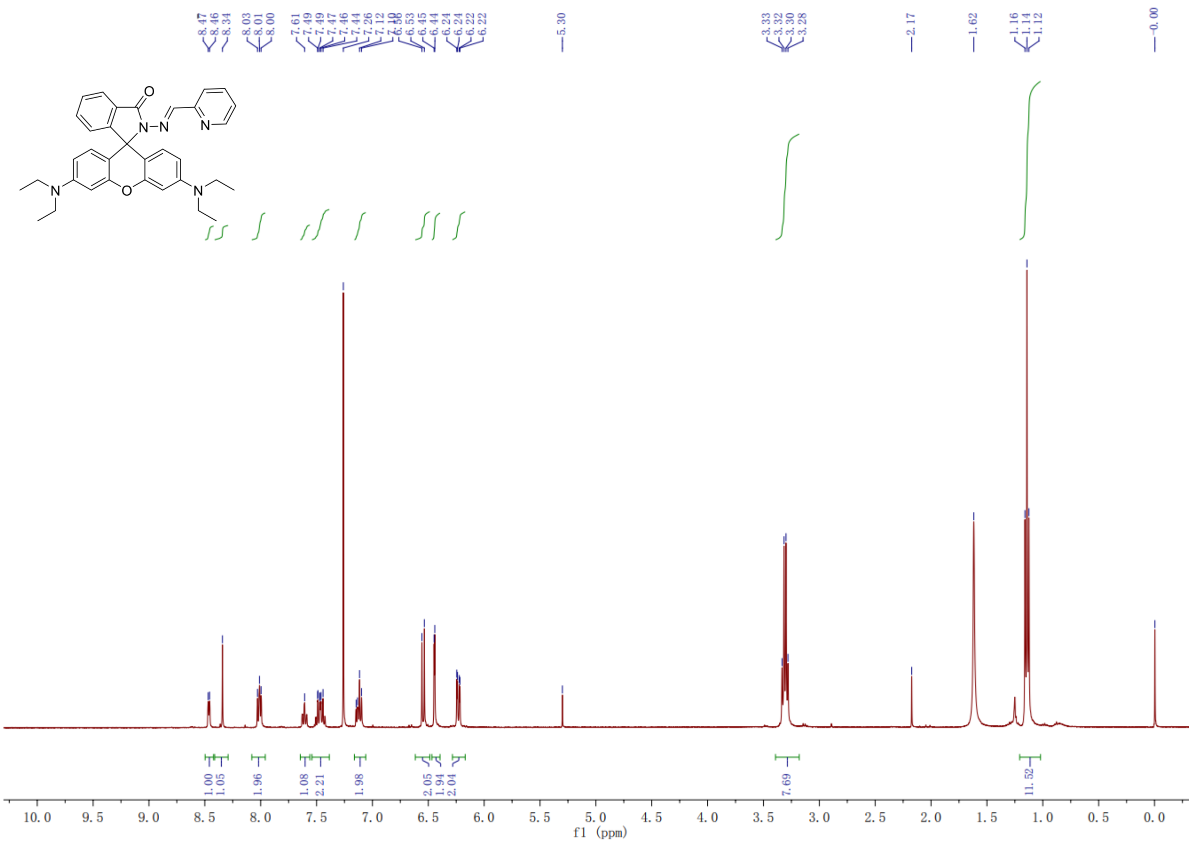

Figure S10. ^{1}H NMR (400 MHz) spectrum of RCO in $CDCl_3$.

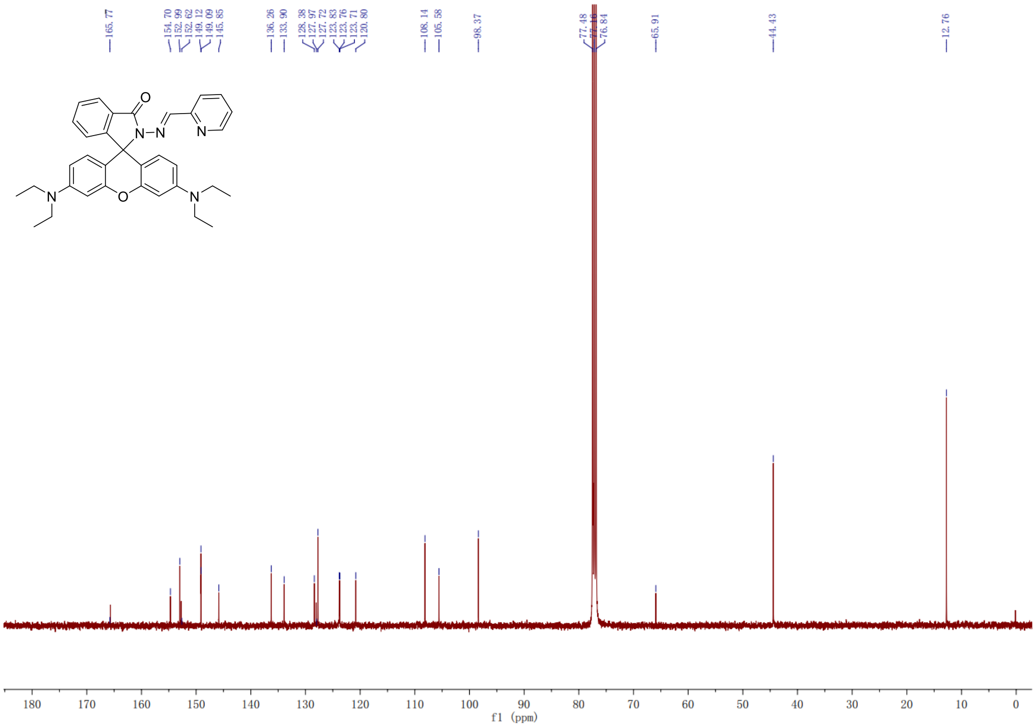

Figure S11. ^{13}C {1H} NMR (101 MHz) spectrum of RCO in $CDCl_3$.

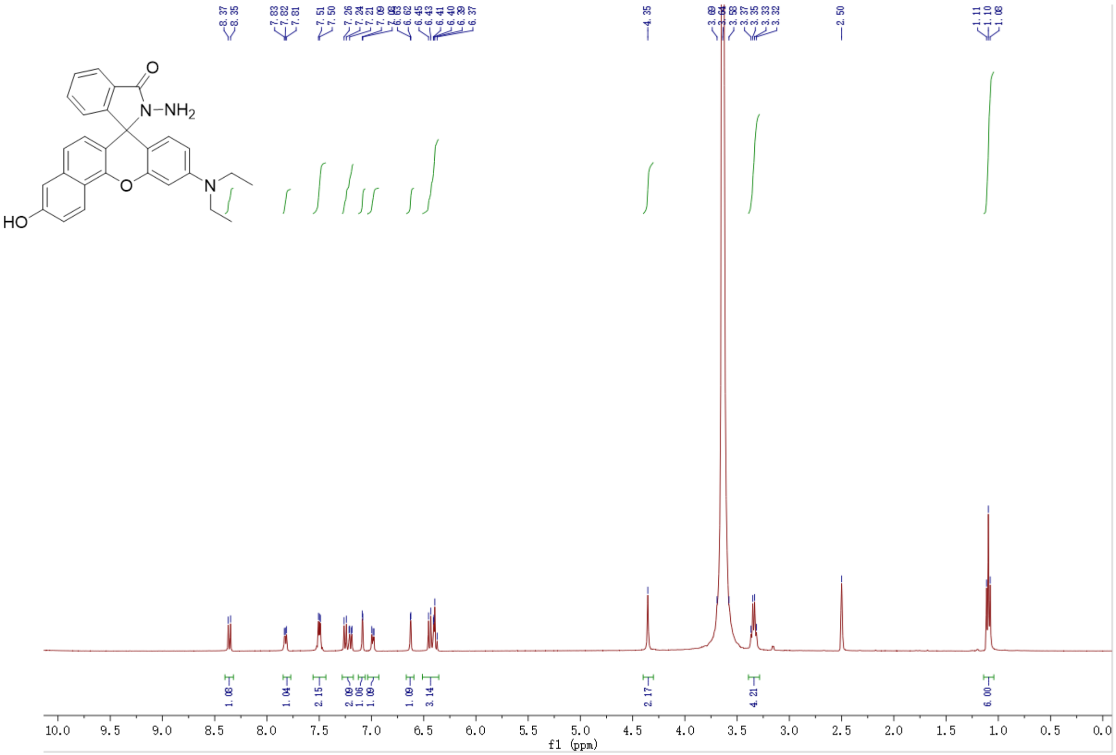

Figure S12. ^{1}H NMR (400 MHz) spectrum of DEB-HY in DMSO-d_6.

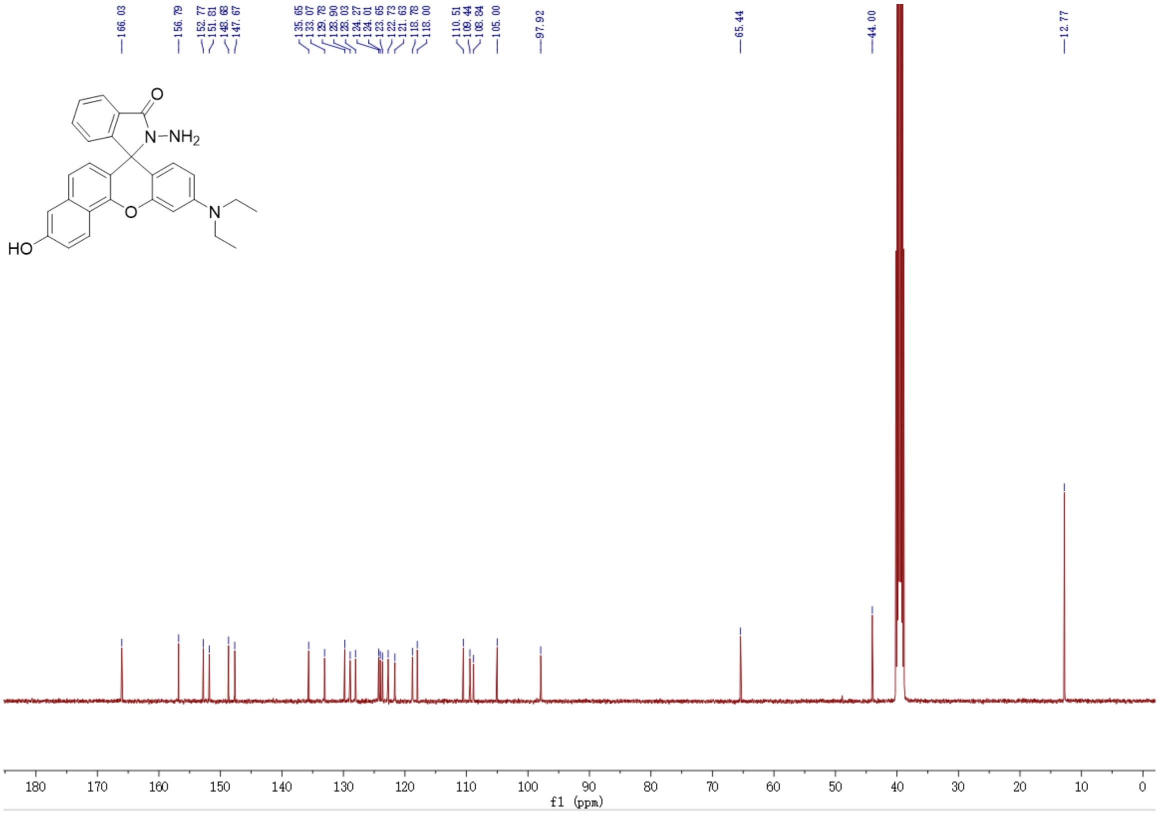

Figure S13. ^{13}C {1H} NMR (101 MHz) spectrum of DEB-HY in DMSO-d_6.

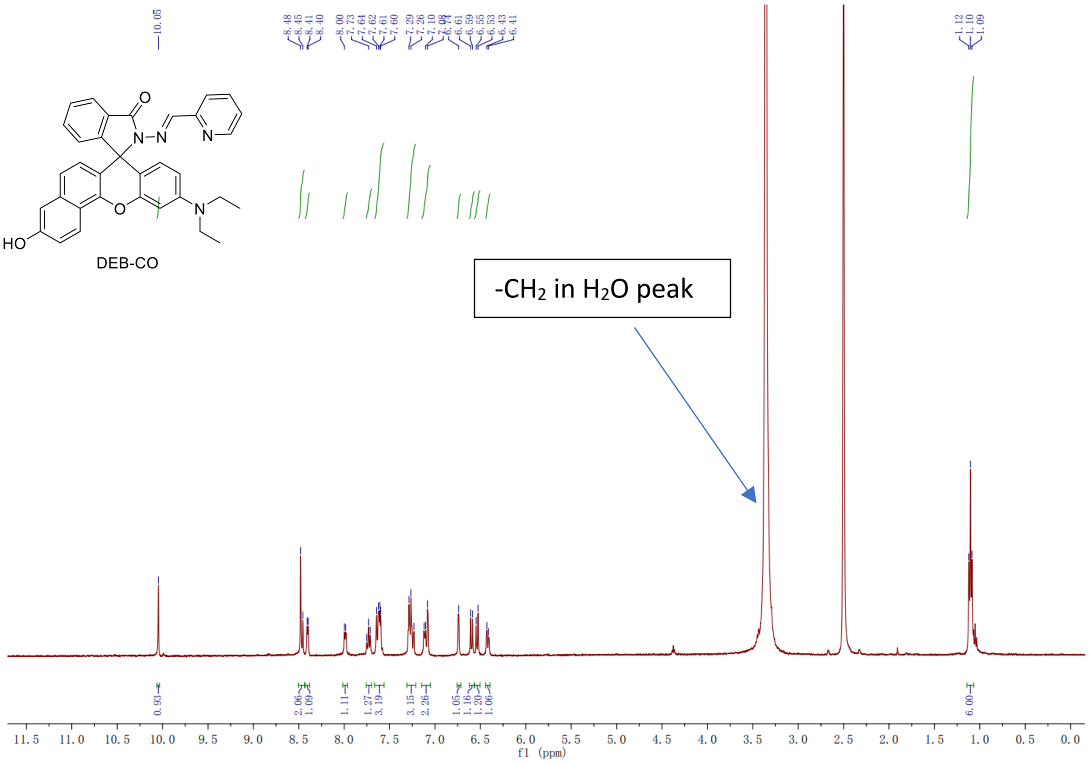

Figure S14. ^{1}H NMR (400 MHz) spectrum of DEB-CO in DMSO-d_6.

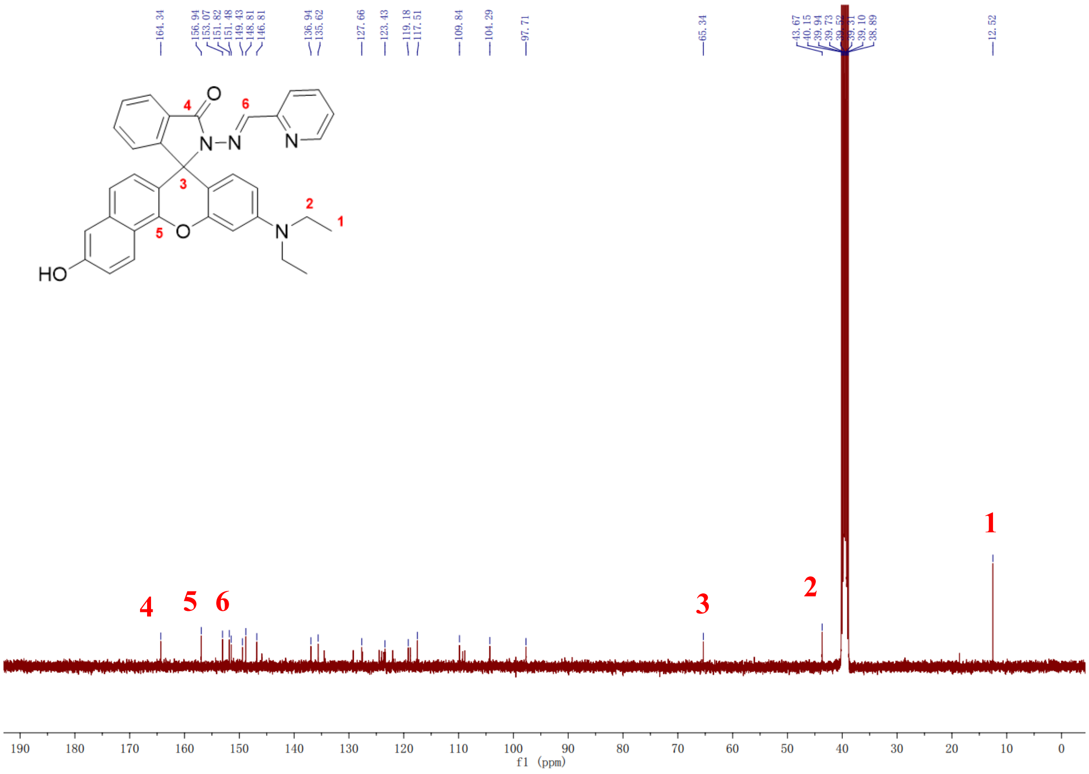

Figure S15. ^{13}C {1H} NMR (101 MHz) spectrum of DEB-CO in DMSO-d_6.

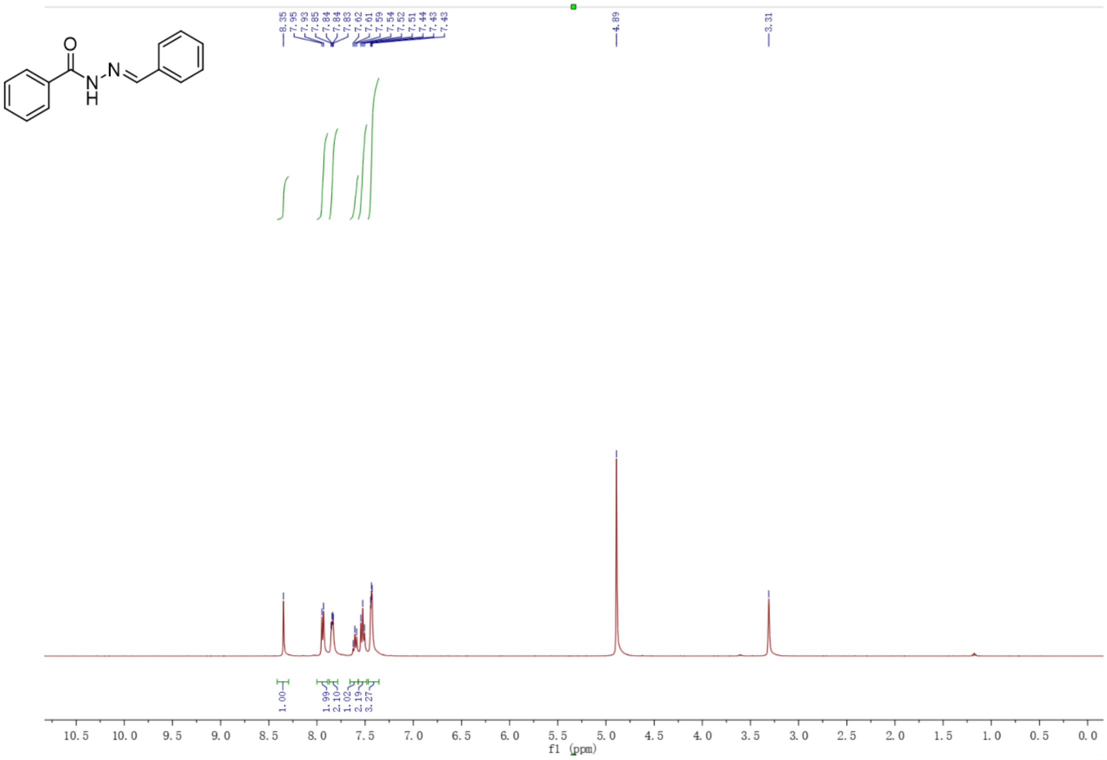

Figure S16. ^{1}H NMR (400 MHz) spectrum of hydrazone compound 1 in MeOD-d_4.

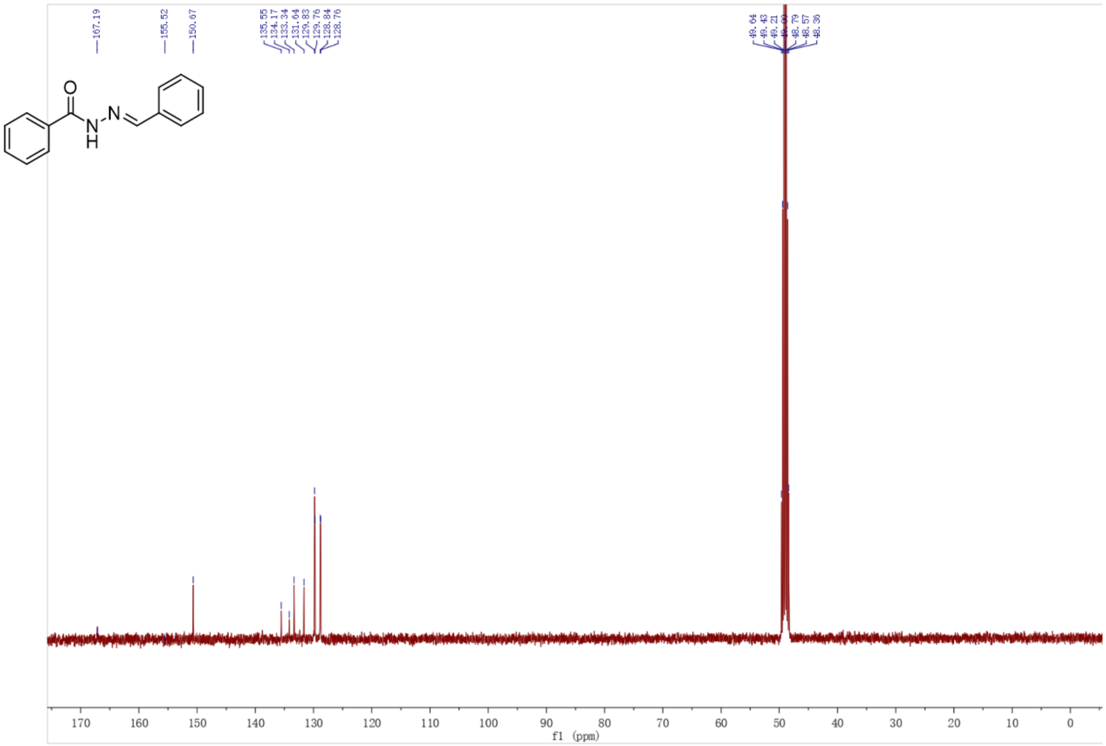

Figure S17. ^{13}C {1H} NMR (101 MHz) spectrum of hydrazone compound 1 in MeOD-d_4.

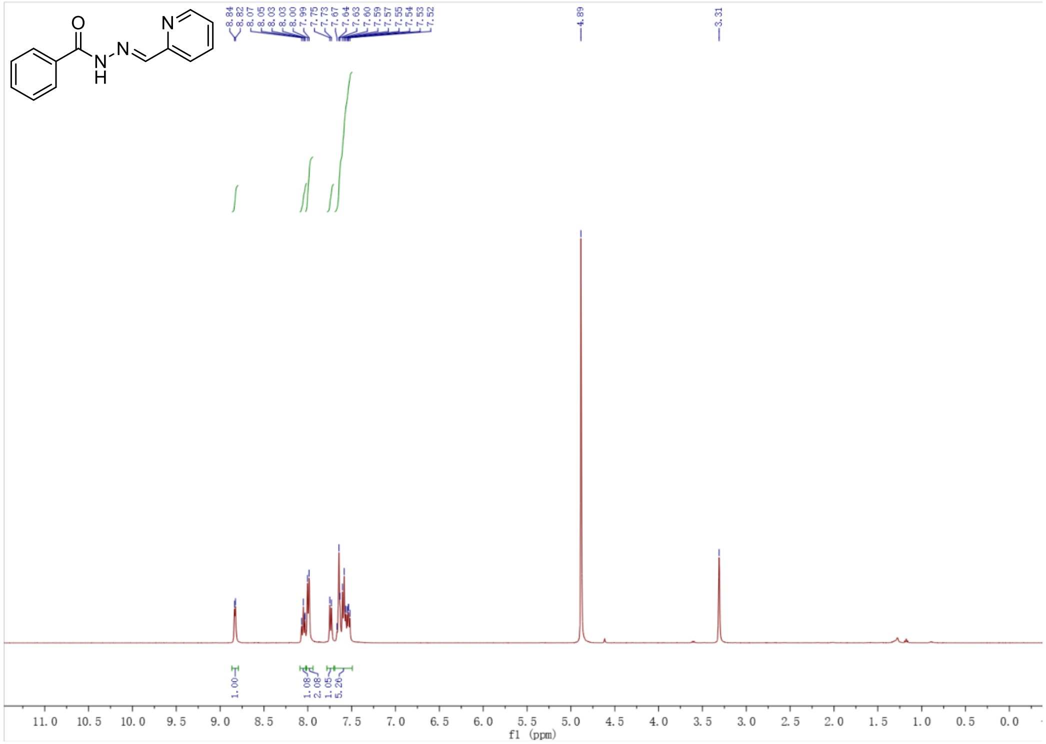

Figure S18. ¹H NMR (400 MHz) spectrum of hydrazone compound 2 in MeOD-d_4.

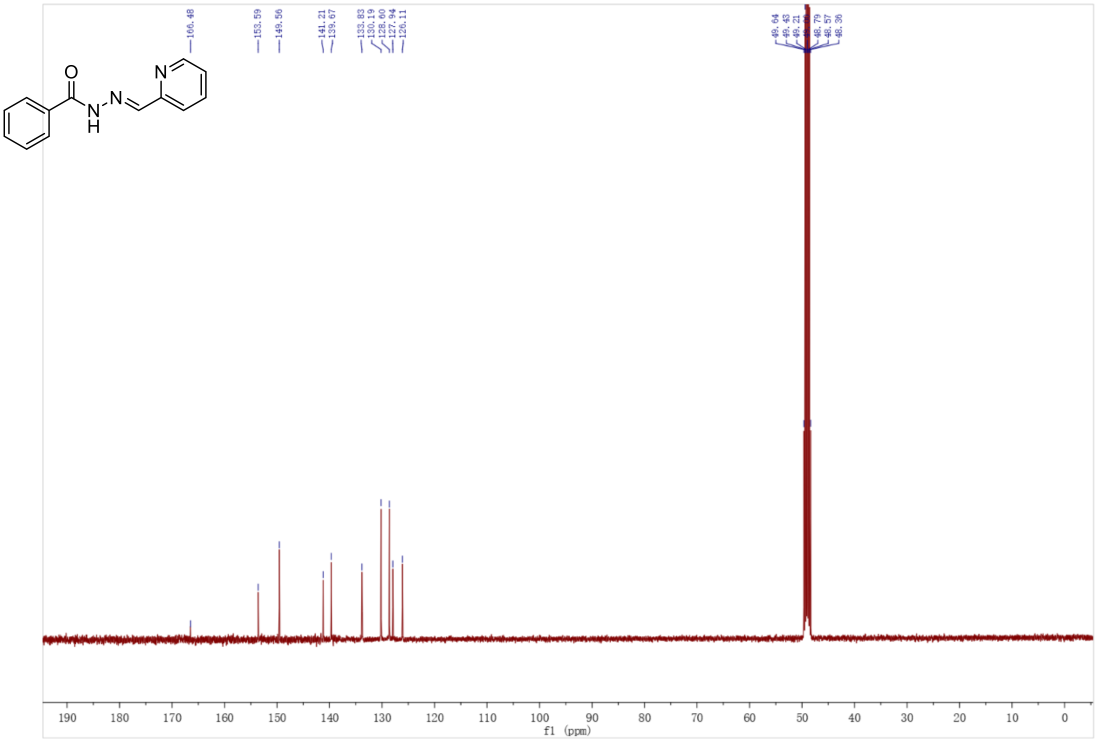

Figure S19. ¹³C {1H} NMR (101 MHz) spectrum of hydrazone compound 2 in MeOD-d_4.

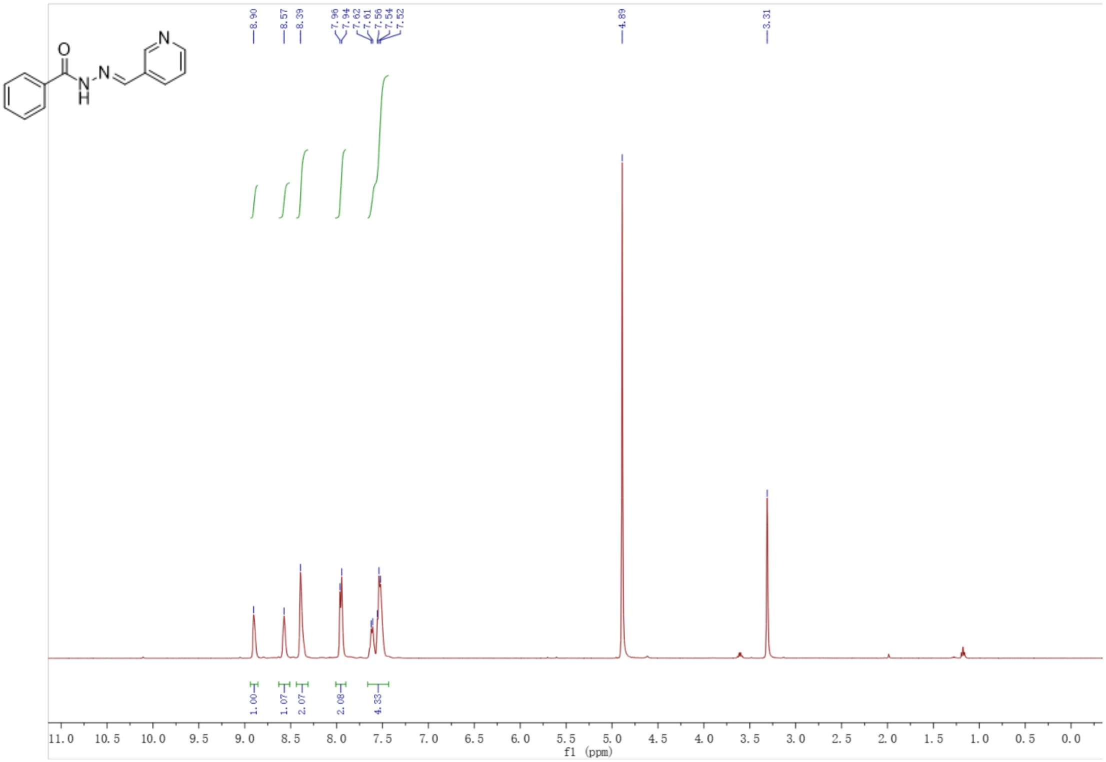

Figure S20. ^{1}H NMR (400 MHz) spectrum of hydrazone compound 3 in MeOD-d_4.

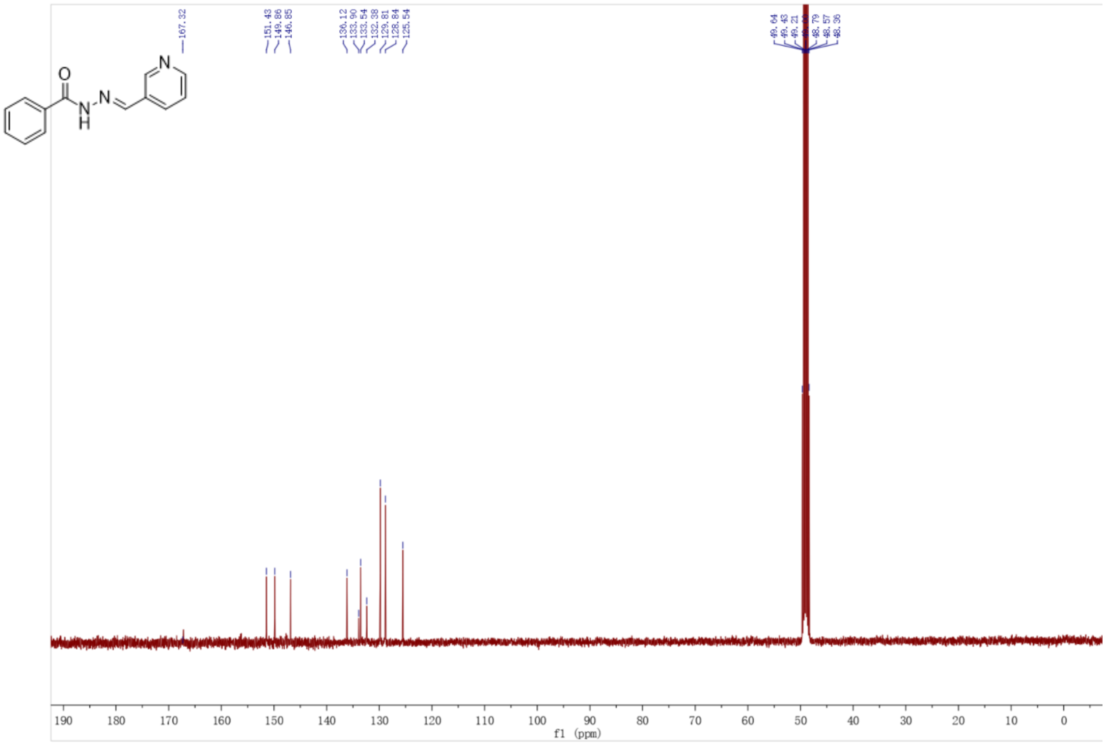

Figure S21. ^{13}C {1H} NMR (101 MHz) spectrum of hydrazone compound 3 in MeOD-d_4.

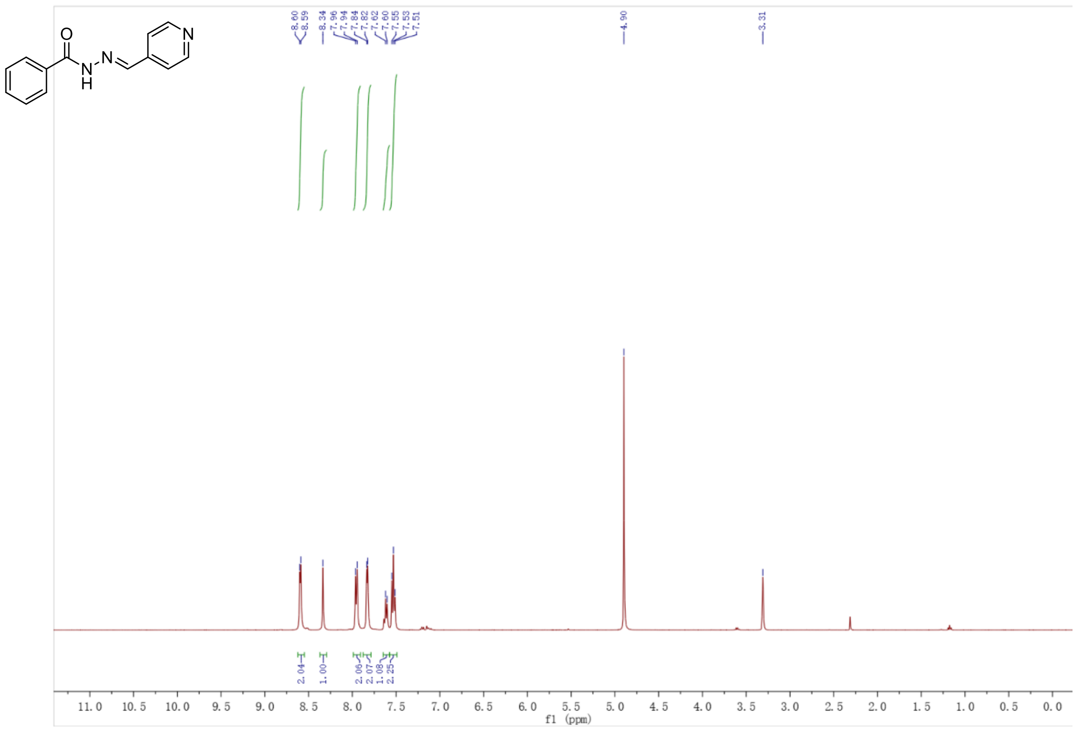

Figure S22. ¹H NMR (400 MHz) spectrum of hydrazone compound 4 in MeOD-d_4.

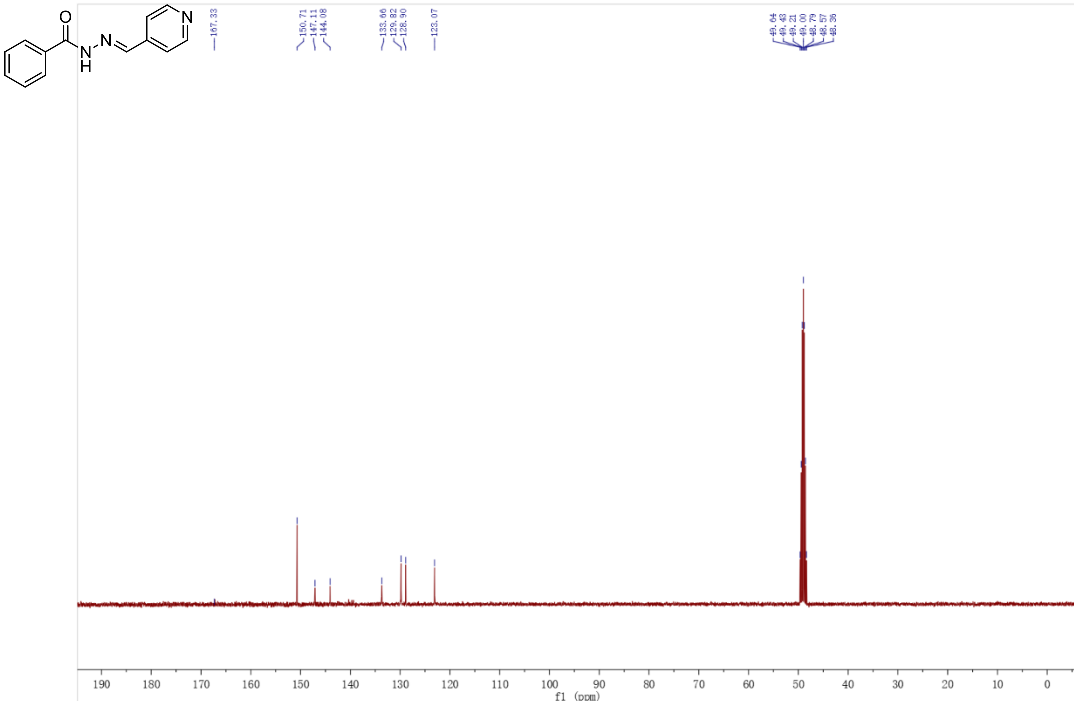

Figure S23. ^{13}C {1H} NMR (101 MHz) spectrum of hydrazone compound 4 in MeOD-d_4.

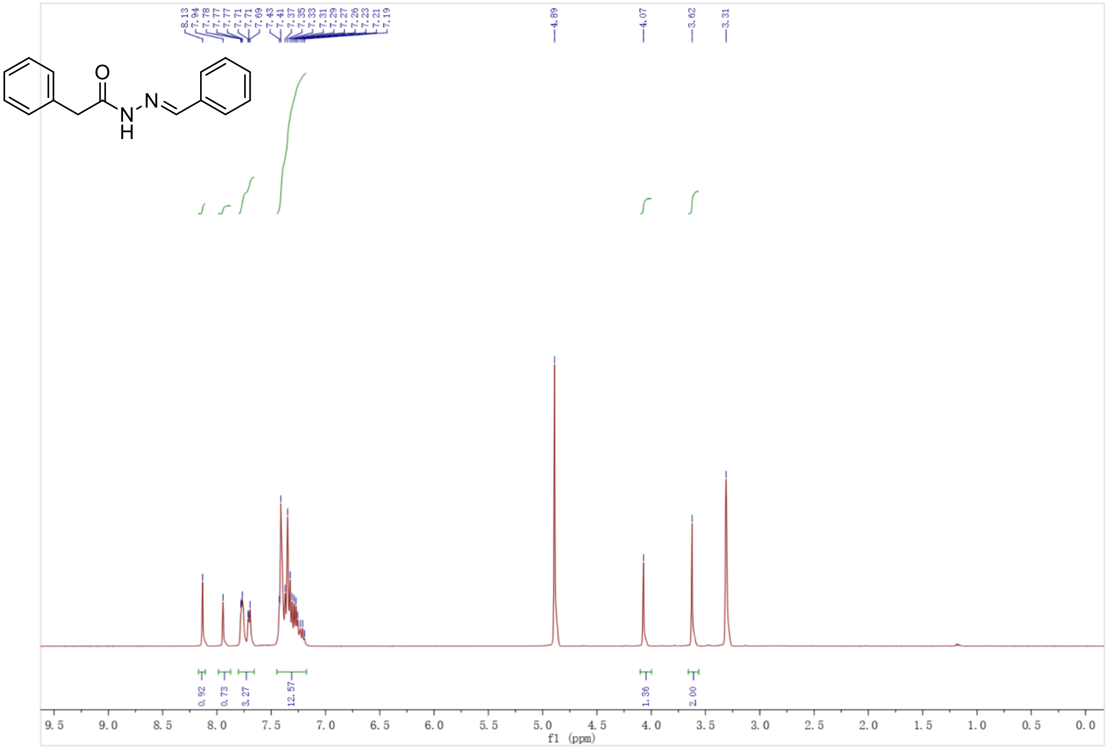

Figure S24. ^{1}H NMR (400 MHz) spectrum of hydrazone compound 5 in MeOD-d_4.

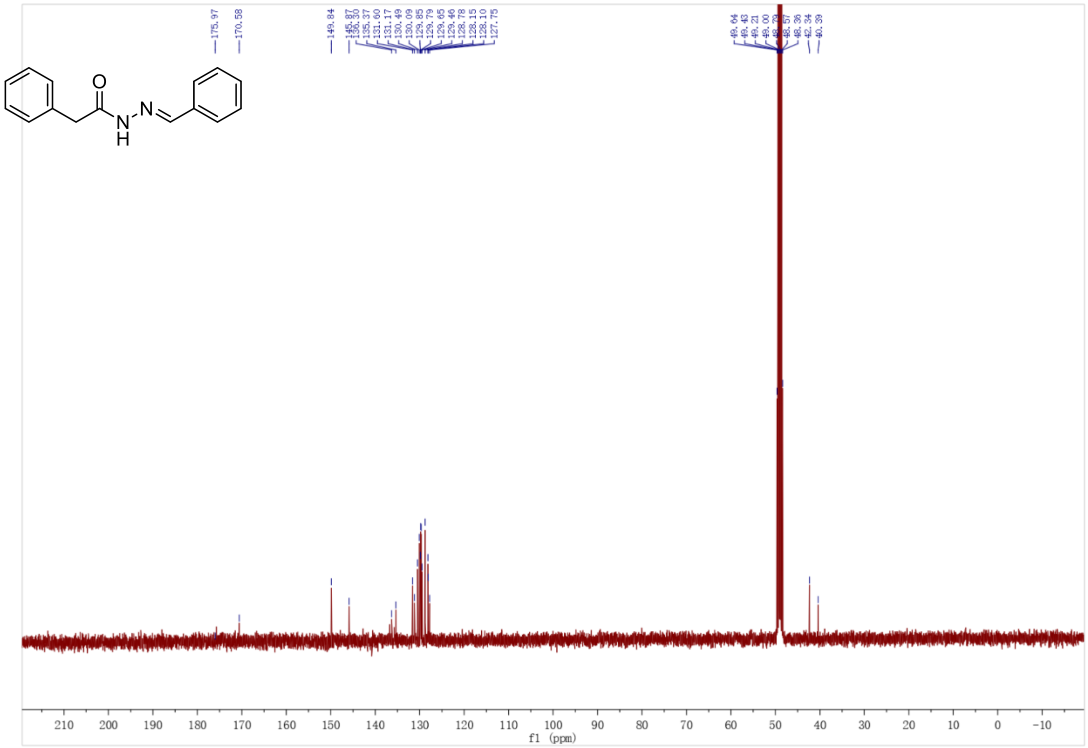

Figure S25. ^{13}C {1H} NMR (101 MHz) spectrum of hydrazone compound 5 in MeOD-d_4.

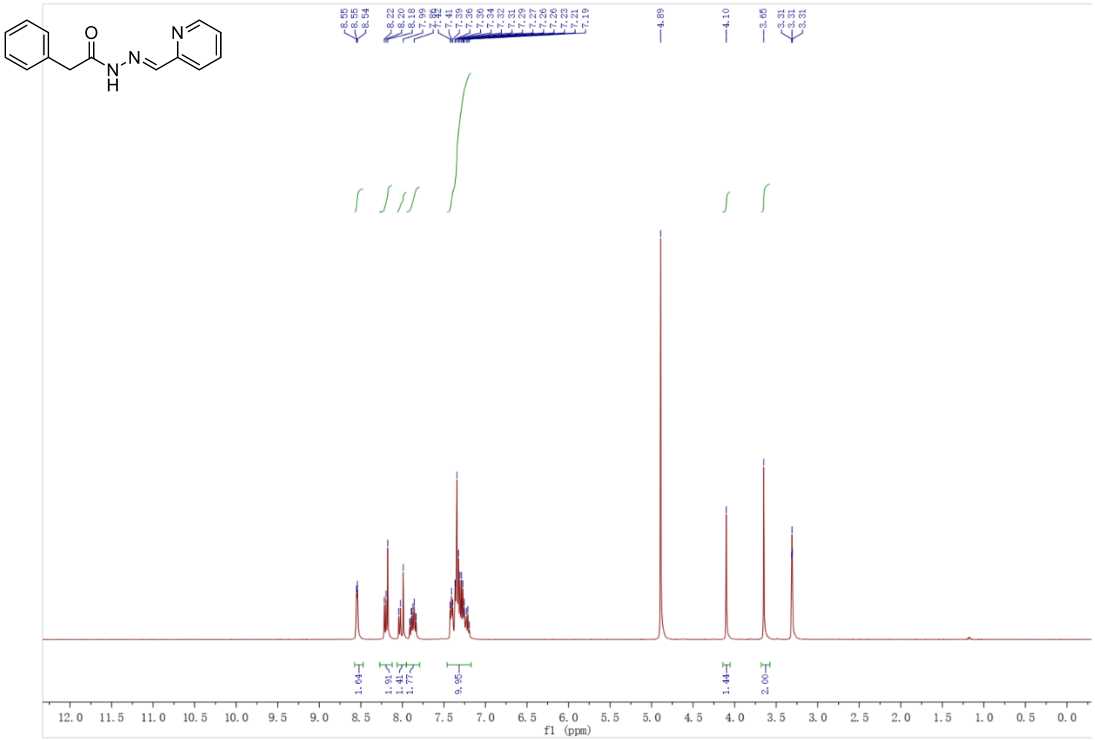

Figure S26. ^{1}H NMR (400 MHz) spectrum of hydrazone compound 6 in MeOD-d_4.

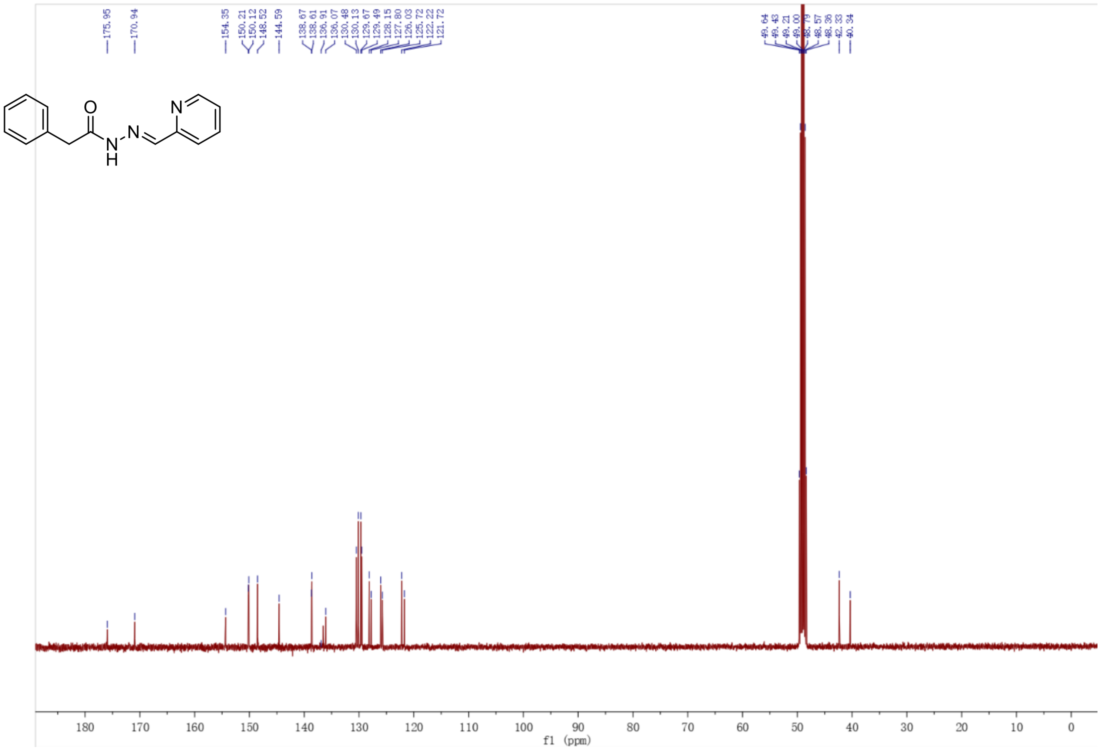

Figure S27. ^{13}C {1H} NMR (101 MHz) spectrum of hydrazone compound 6 in MeOD-d_4.

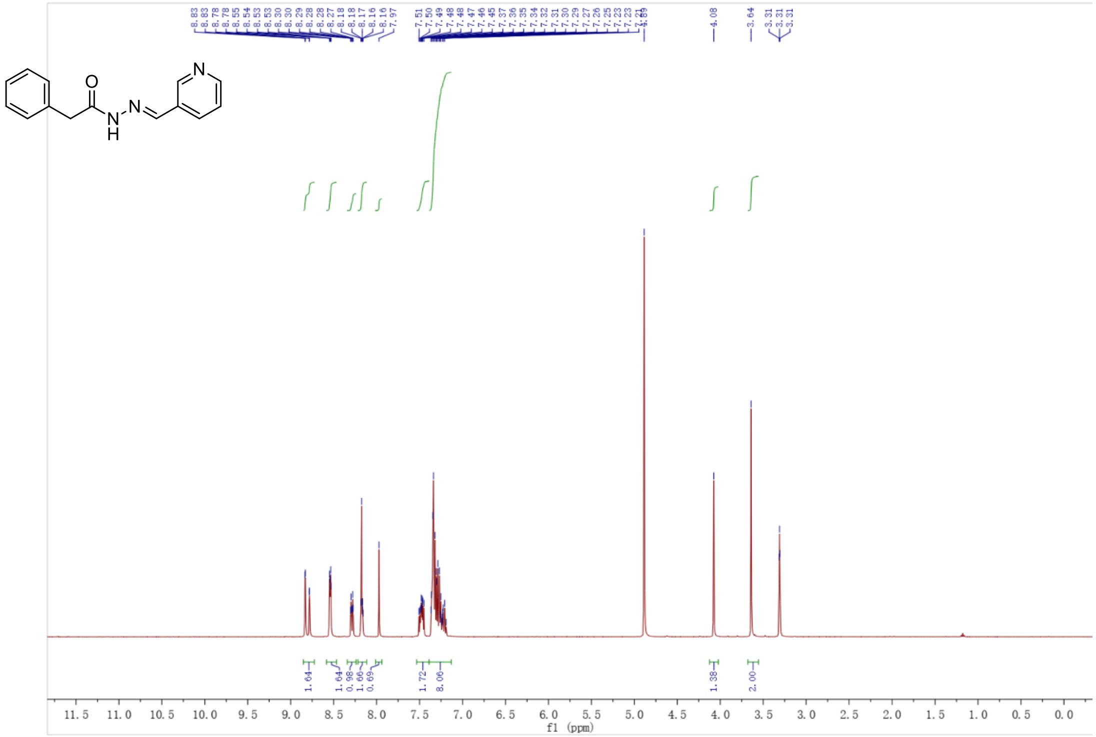

Figure S28. ^{1}H NMR (400 MHz) spectrum of hydrazone compound 7 in MeOD-d_4.

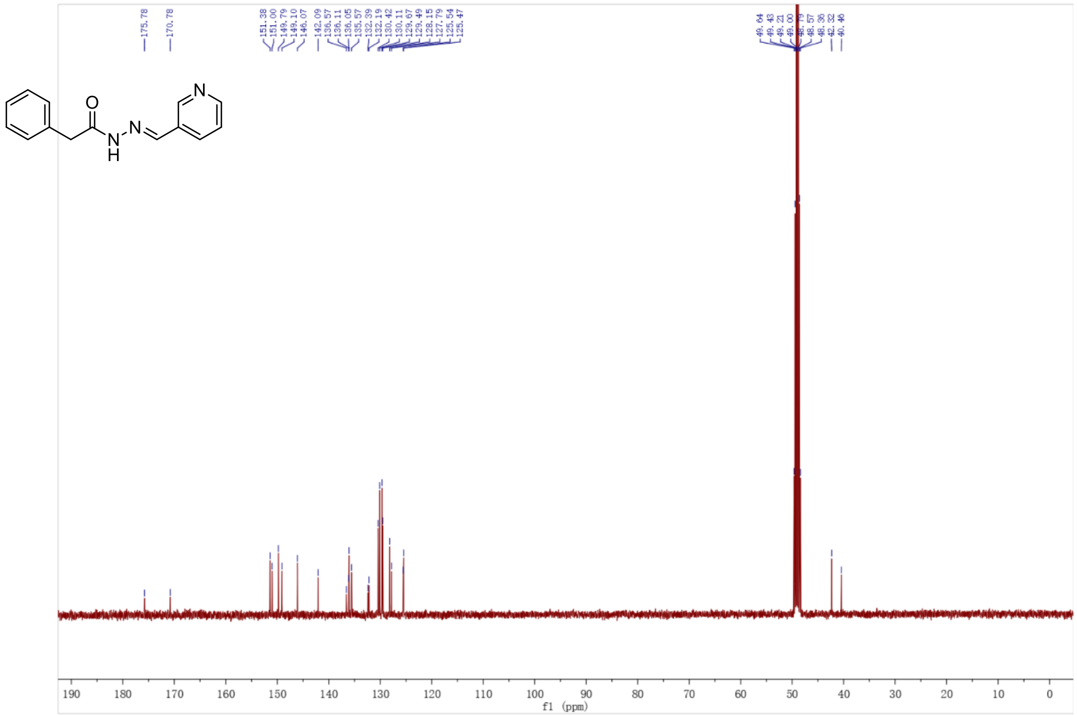

Figure S29. ^{13}C {1H} NMR (101 MHz) spectrum of hydrazone compound 7 in MeOD-d_4.

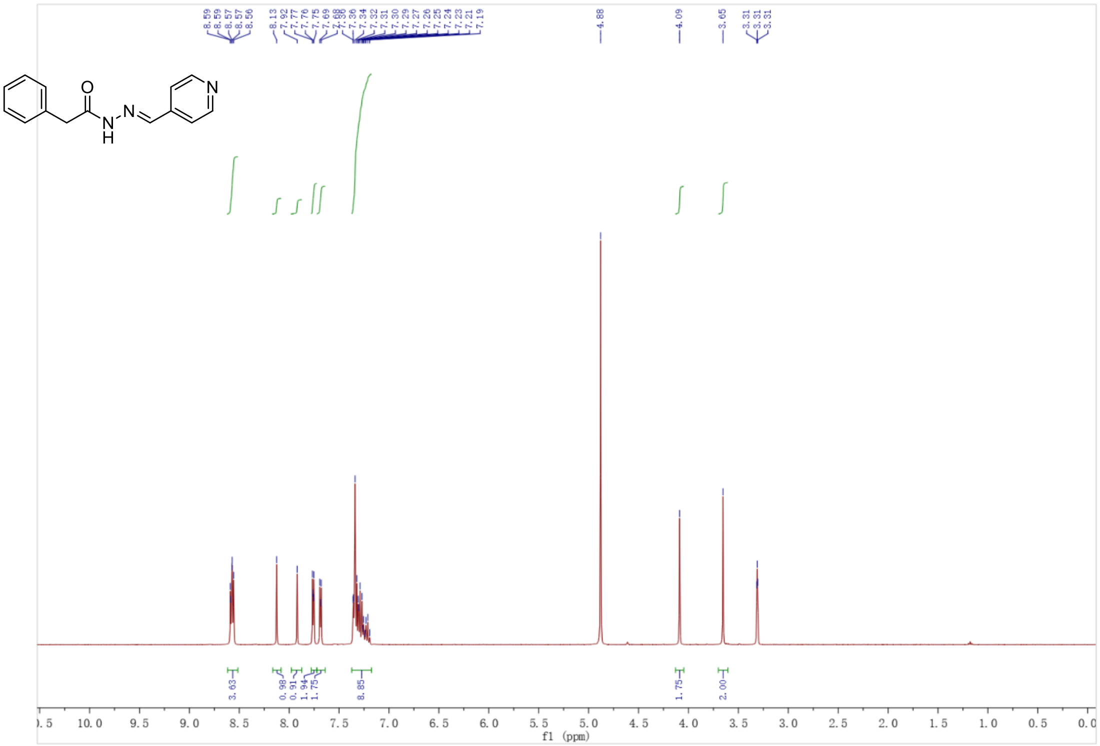

Figure S30. ^{1}H NMR (400 MHz) spectrum of hydrazone compound 8 in MeOD-d_4.

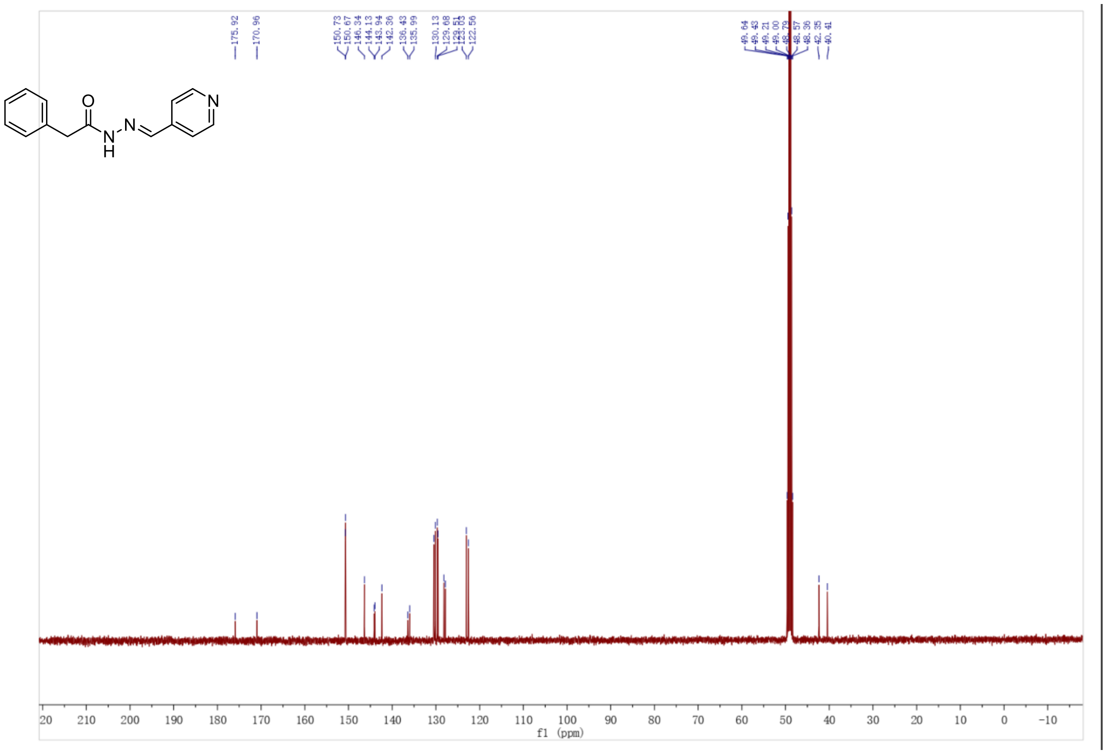

Figure S31. ^{13}C {1H} NMR (101 MHz) spectrum of hydrazone compound 8 in MeOD-d_4.

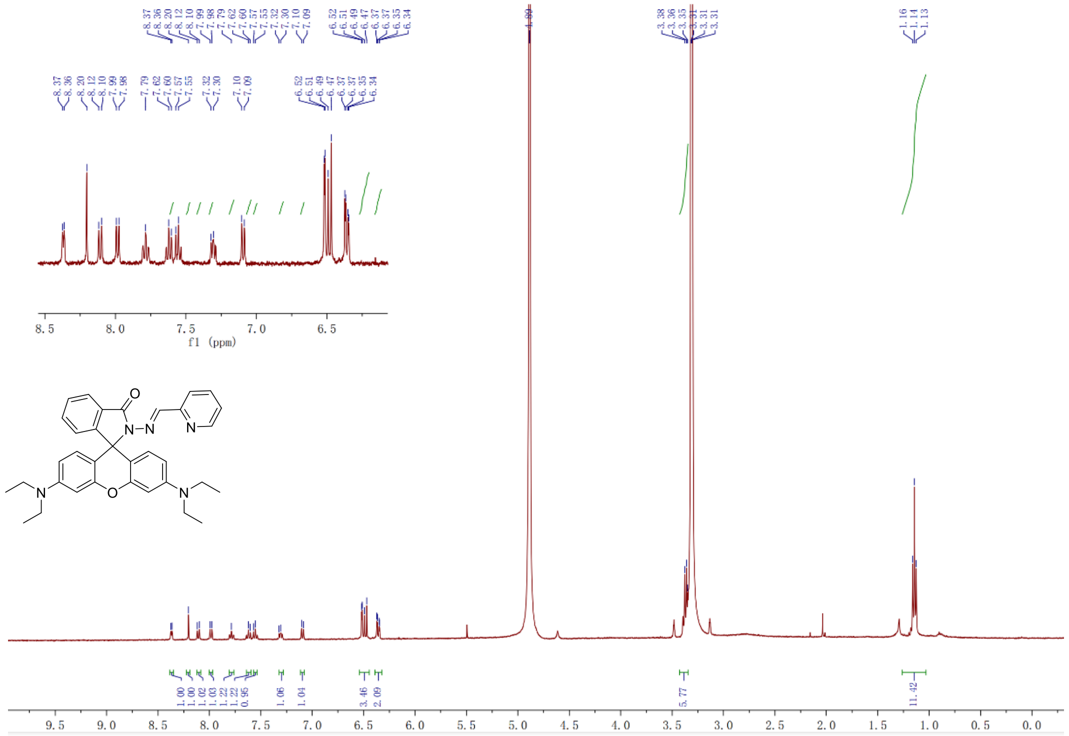

Figure S32. ^{1}H NMR (400 MHz) spectrum of 1 mM RCO in MeOD-d_4.

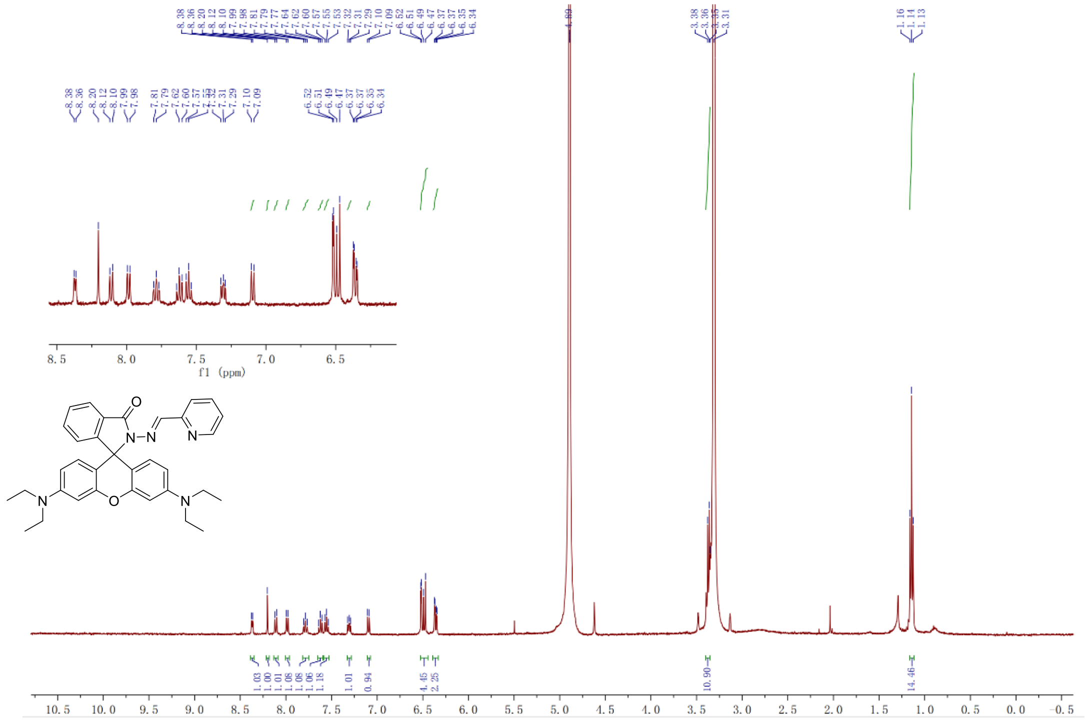

Figure S33. ^{1}H NMR (400 MHz) spectrum of 1 mM RCO in MeOD-d_4 after bubbled CO and incubation.

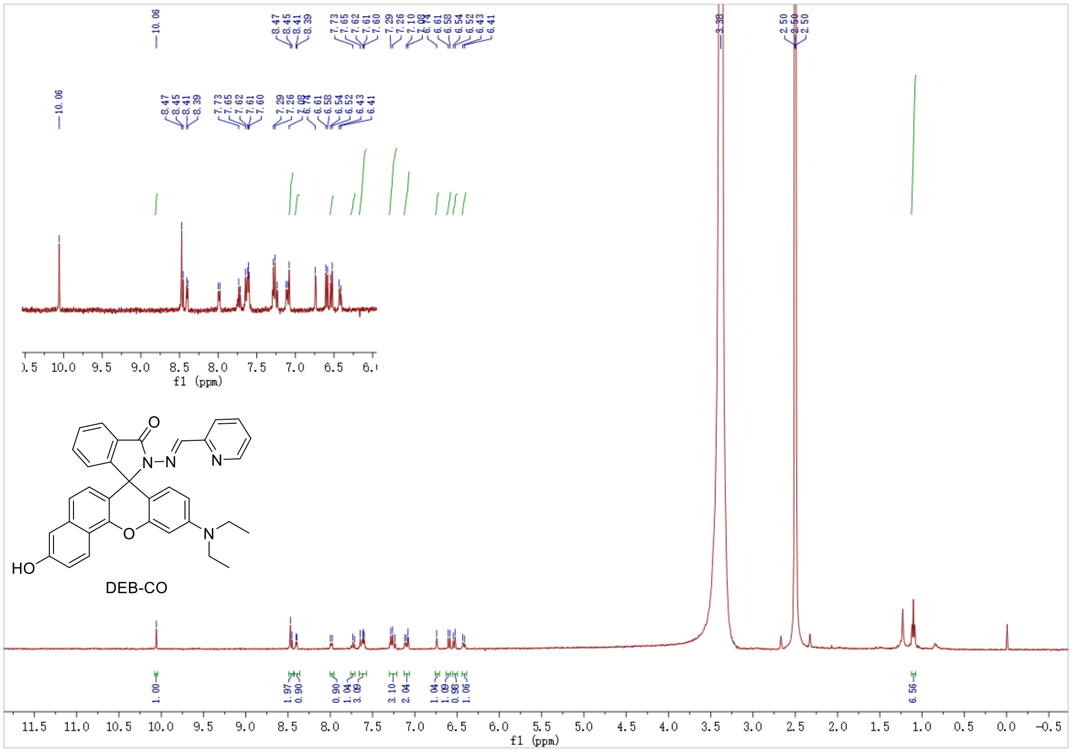

Figure S34. ^{1}H NMR (400 MHz) spectrum of 1 mM DEB-CO in MeOD-d_4.

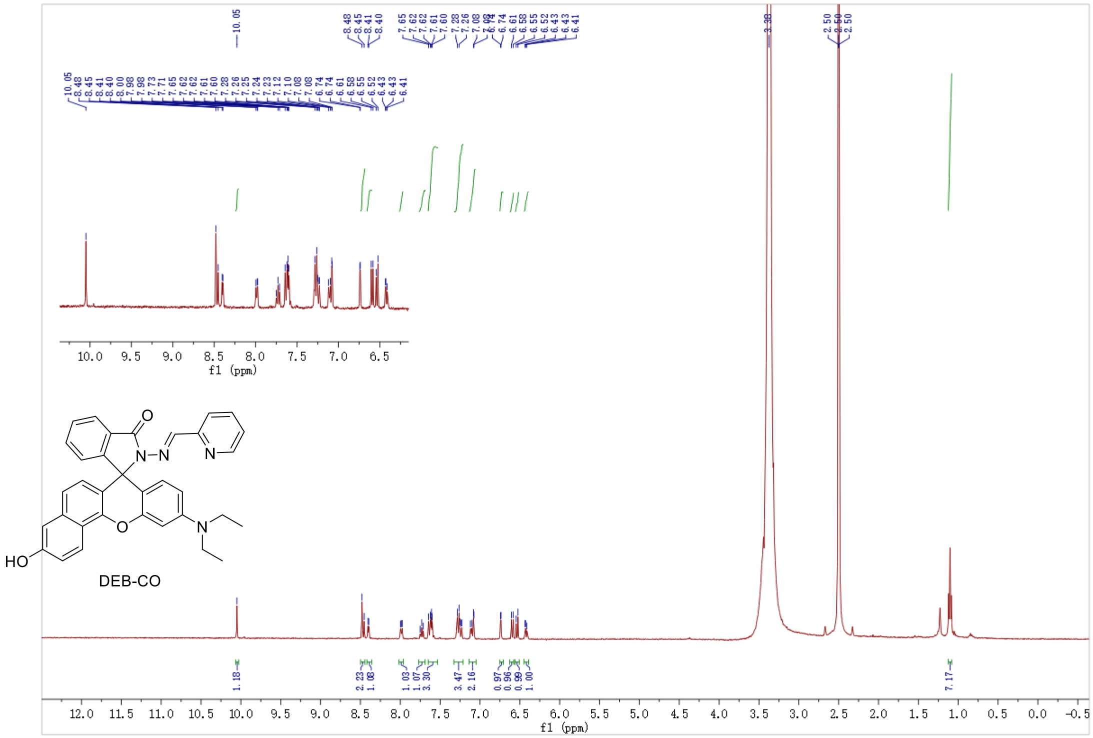

Figure S35. ^{1}H NMR (400 MHz) spectrum of 1 mM DEB-CO in MeOD-d_4 after bubbled CO and incubation.

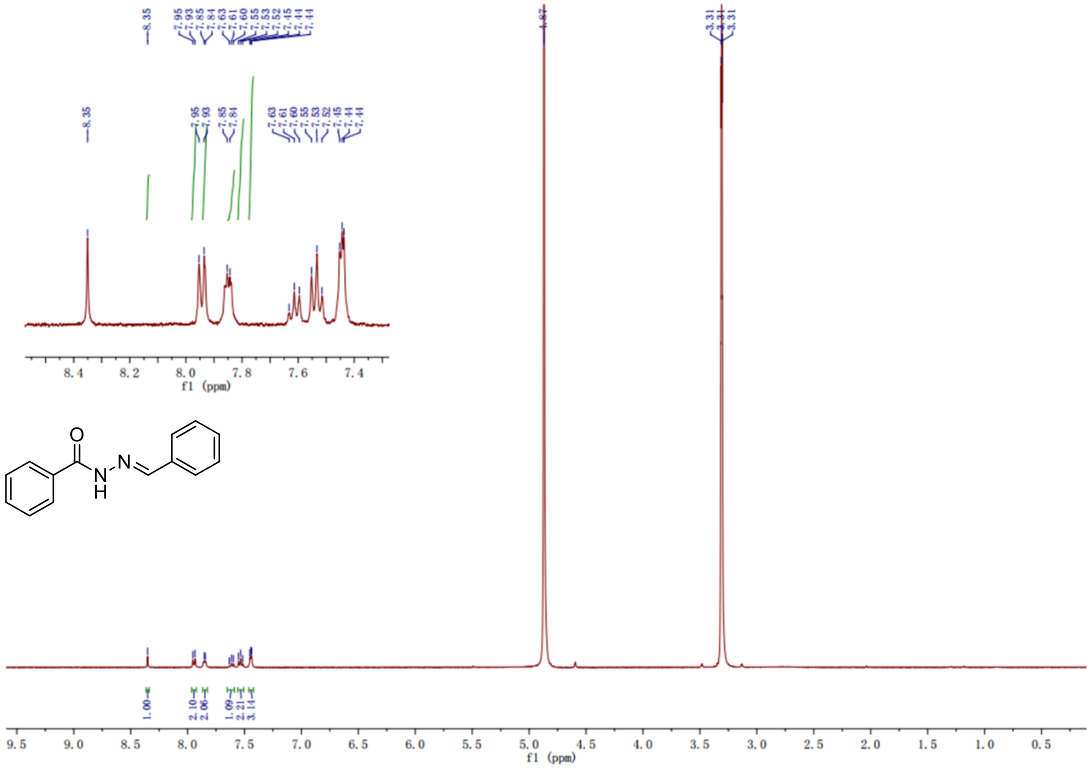

Figure S36. ^{1}H (400 MHz) NMR spectrum of 1 mM hydrazone compound 1 in MeOD-d_4.

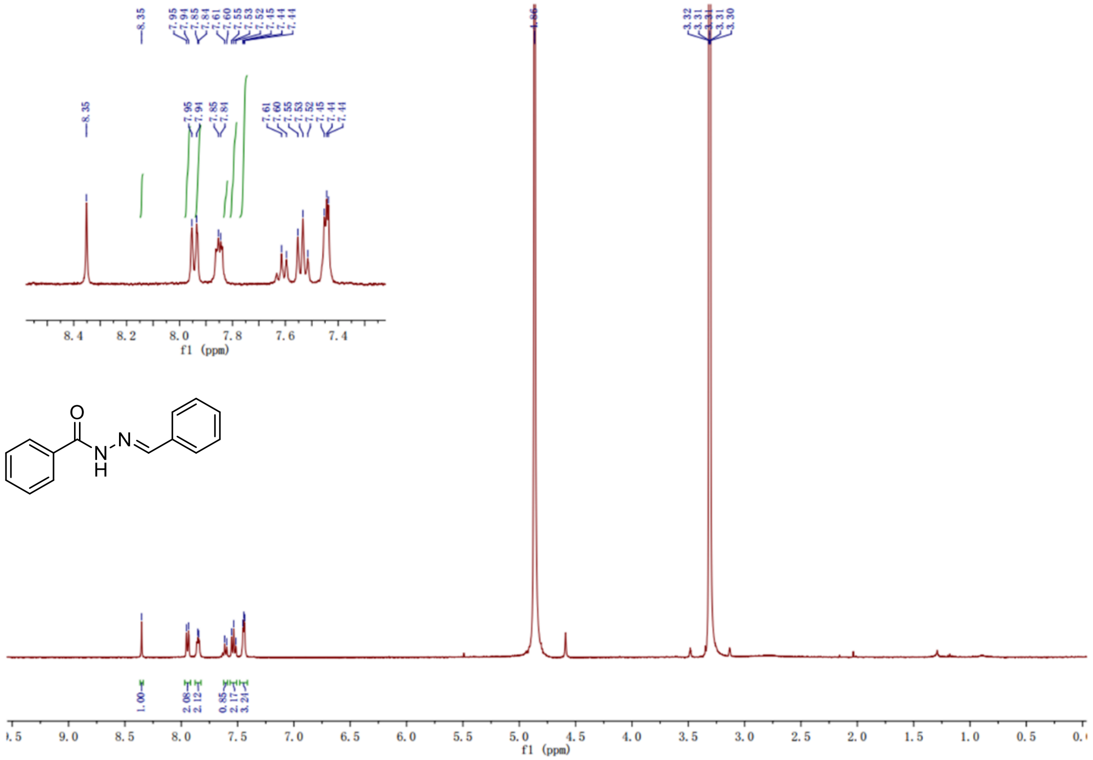

Figure S37. ^{1}H NMR (400 MHz) spectrum of 1 mM hydrazone compound 1 in MeOD-d_4 after bubbled CO and incubation.

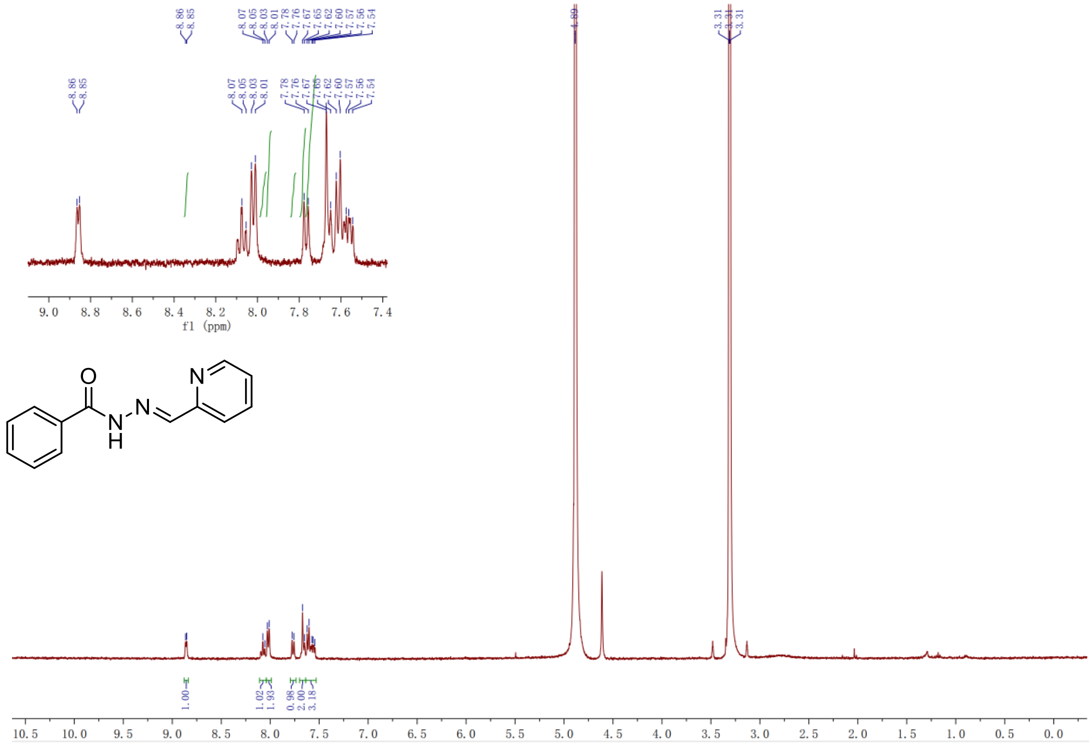

Figure S38. ^{1}H NMR (400 MHz) spectrum of 1 mM hydrazone compound 2 in MeOD-d_4.

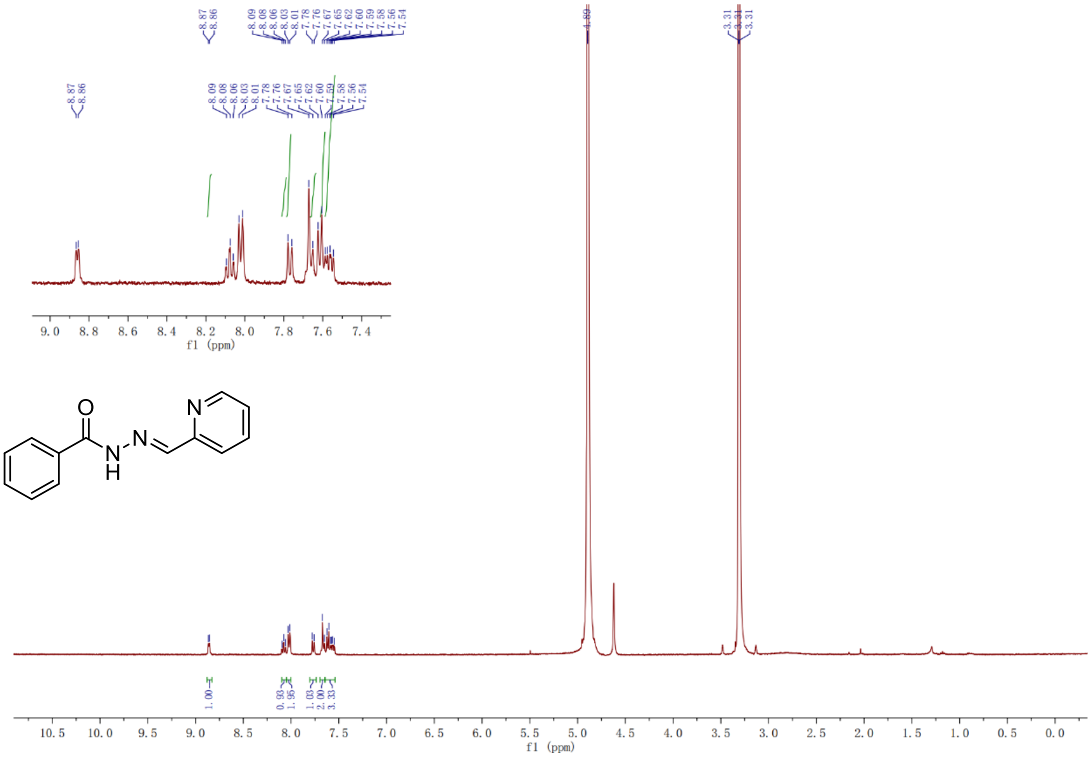

Figure S39. ^{1}H NMR (400 MHz) spectrum of 1 mM hydrazone compound 2 in MeOD-d_4 after bubbled CO and incubation.

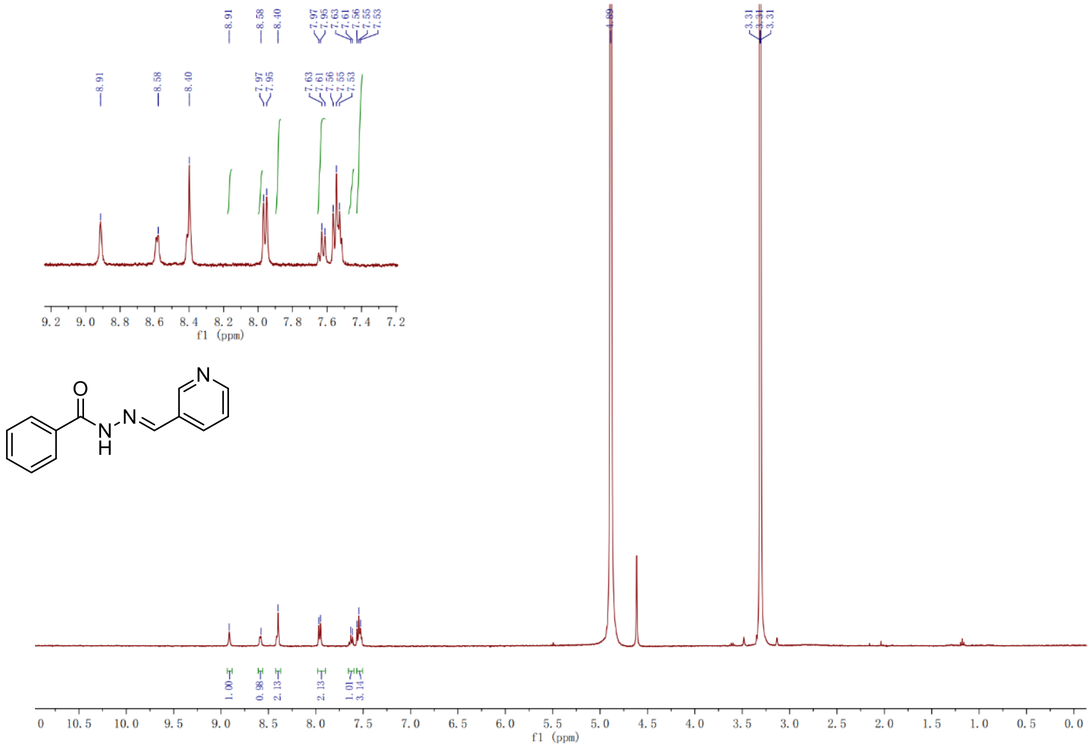

Figure S40. ^{1}H NMR (400 MHz) spectrum of 1 mM hydrazone compound 3 in MeOD-d_4.

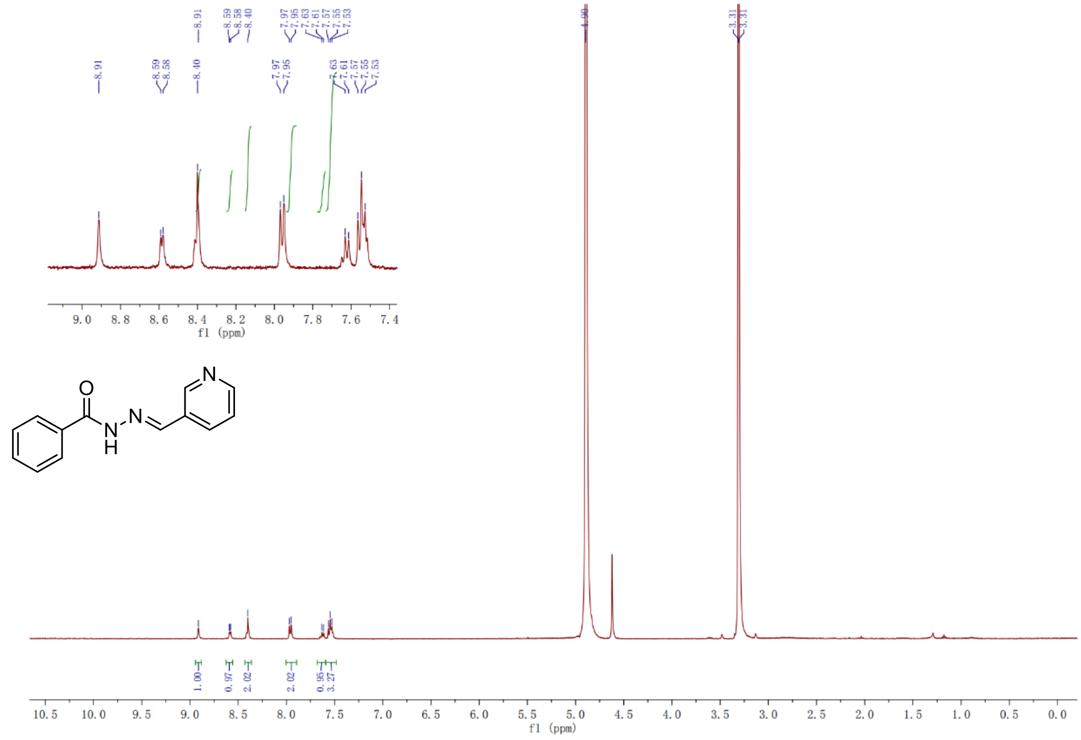

Figure S41. ^{1}H NMR (400 MHz) spectrum of 1 mM hydrazone compound 3 in MeOD-d_4 after bubbled CO and incubation.

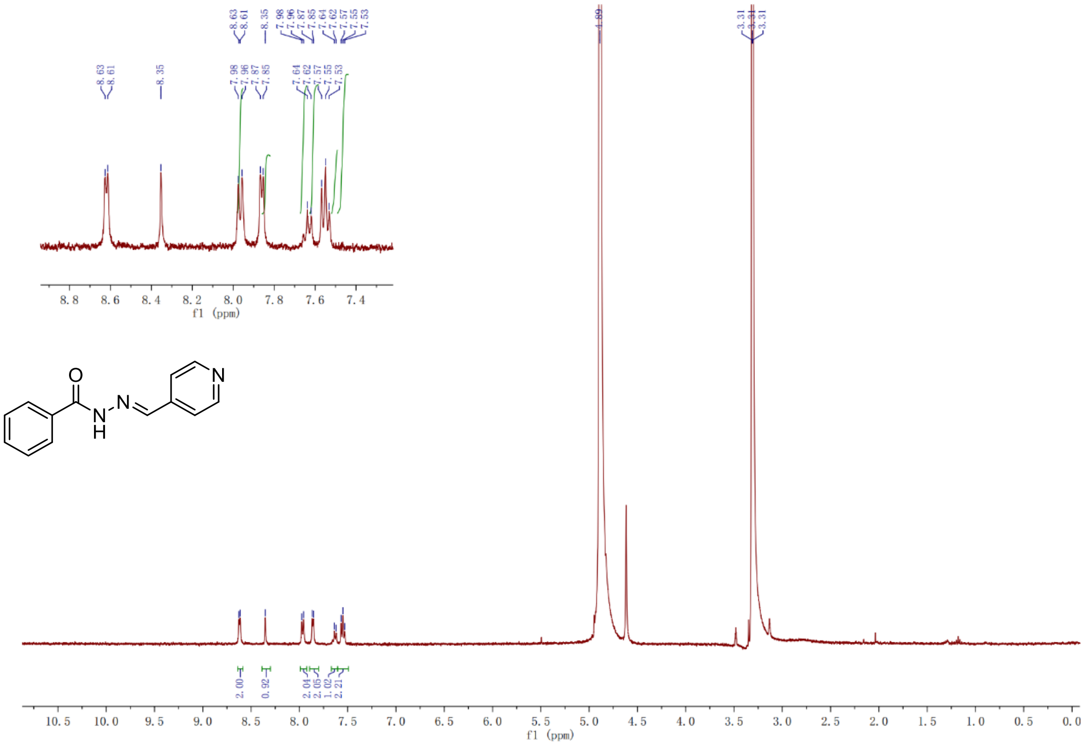

Figure S42. ^{1}H NMR (400 MHz) spectrum of 1 mM hydrazone compound 4 in MeOD-d_4.

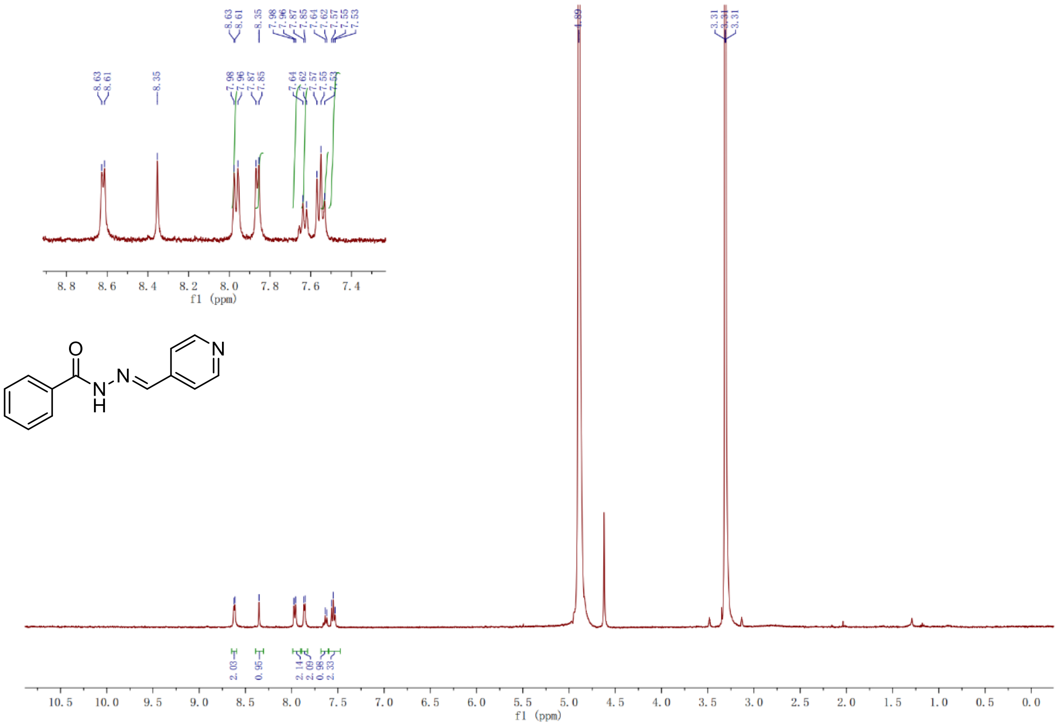

Figure S43. ^{1}H NMR (400 MHz) spectrum of 1 mM hydrazone compound 4 in MeOD-d_4 after bubbled CO and incubation.

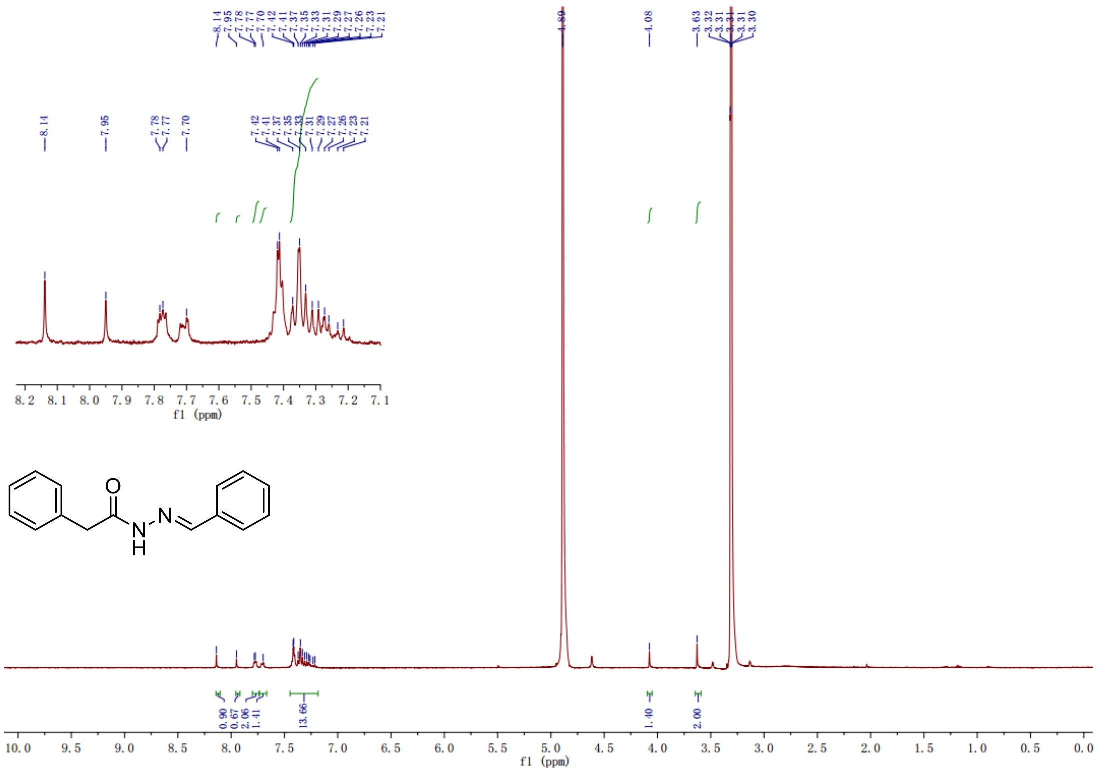

Figure S44. ^{1}H NMR (400 MHz) spectrum of 1 mM hydrazone compound 5 in MeOD-d_4.

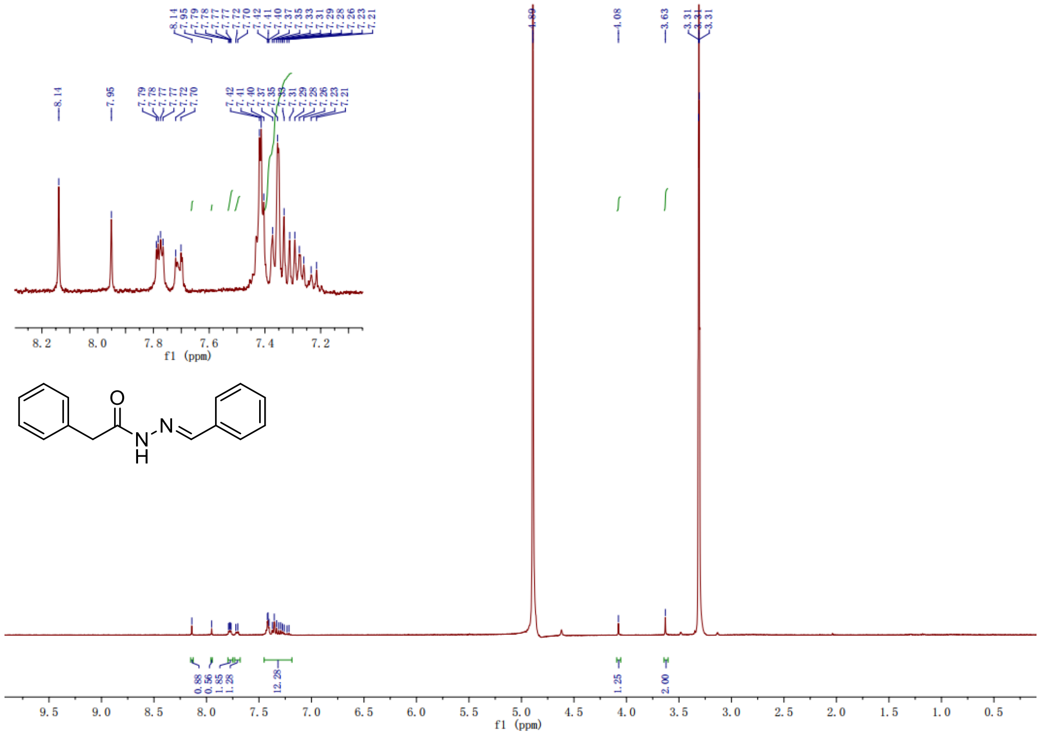

Figure S45. ^{1}H NMR (400 MHz) spectrum of 1 mM hydrazone compound 5 in MeOD-d_4 after bubbled CO and incubation.

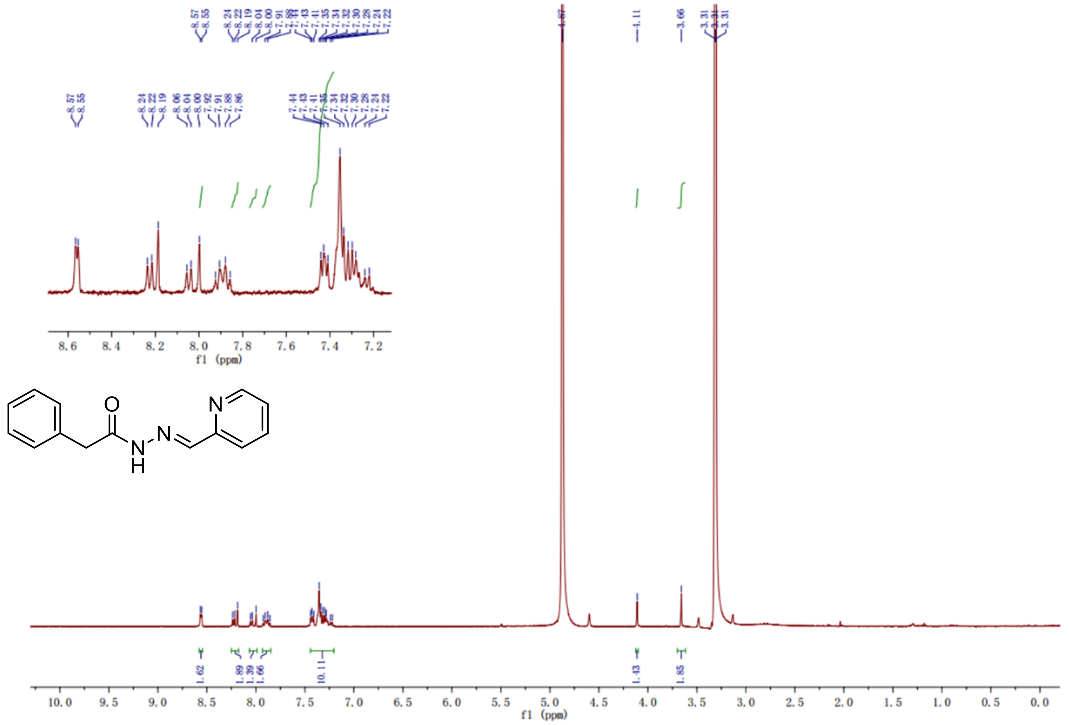

Figure S46. ^{1}H NMR (400 MHz) spectrum of 1 mM hydrazone compound 6 in MeOD-d_4.

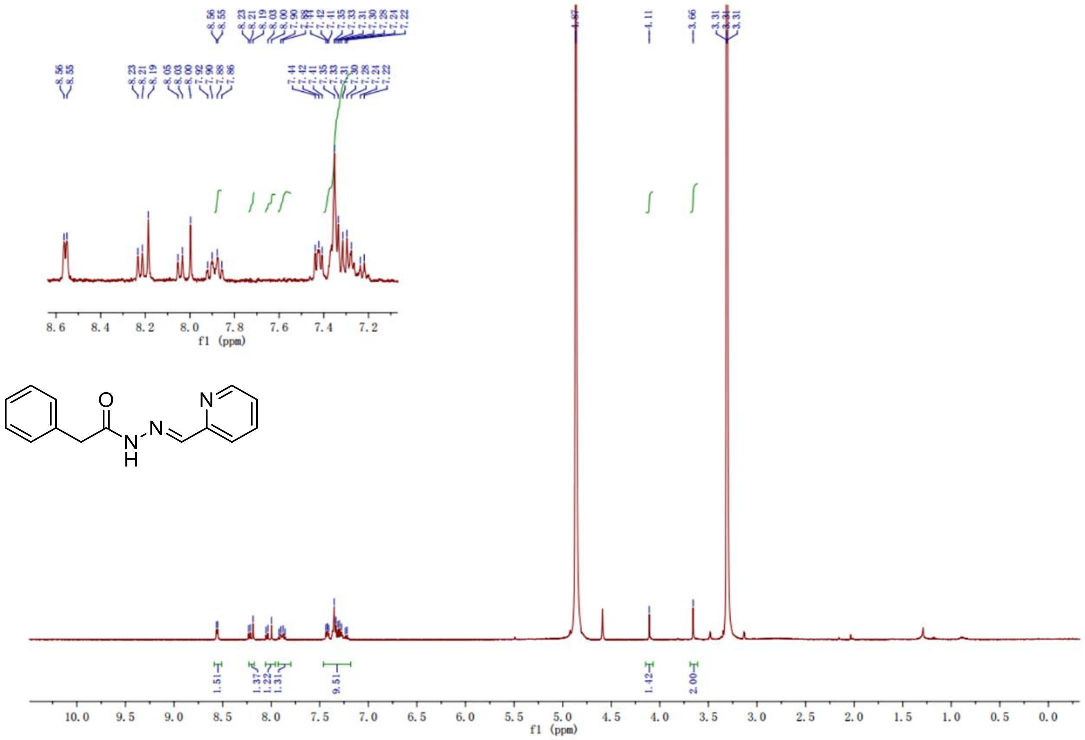

Figure S47. ^{1}H NMR (400 MHz) spectrum of 1 mM hydrazone compound 6 in MeOD-d_4 after bubbled CO and incubation.

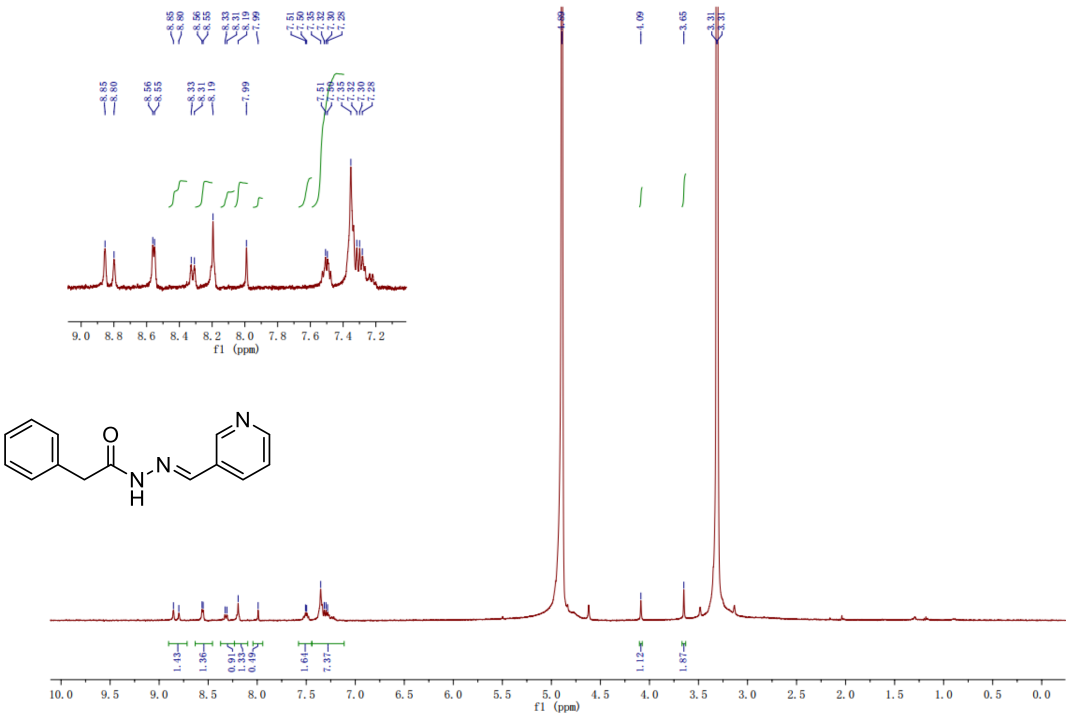

Figure S48. ^{1}H NMR (400 MHz) spectrum of 1 mM hydrazone compound 7 in MeOD-d_4.

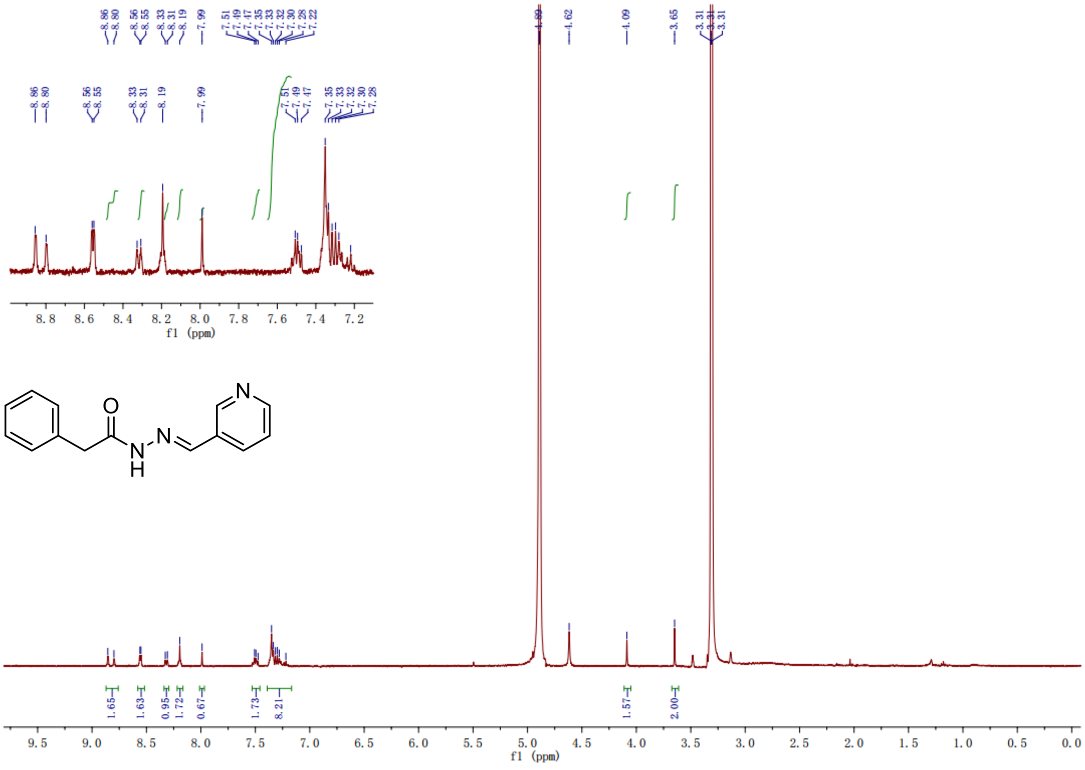

Figure S49. ^{1}H NMR (400 MHz) spectrum of 1 mM hydrazone compound 7 in MeOD-d_4 after bubbled CO and incubation.

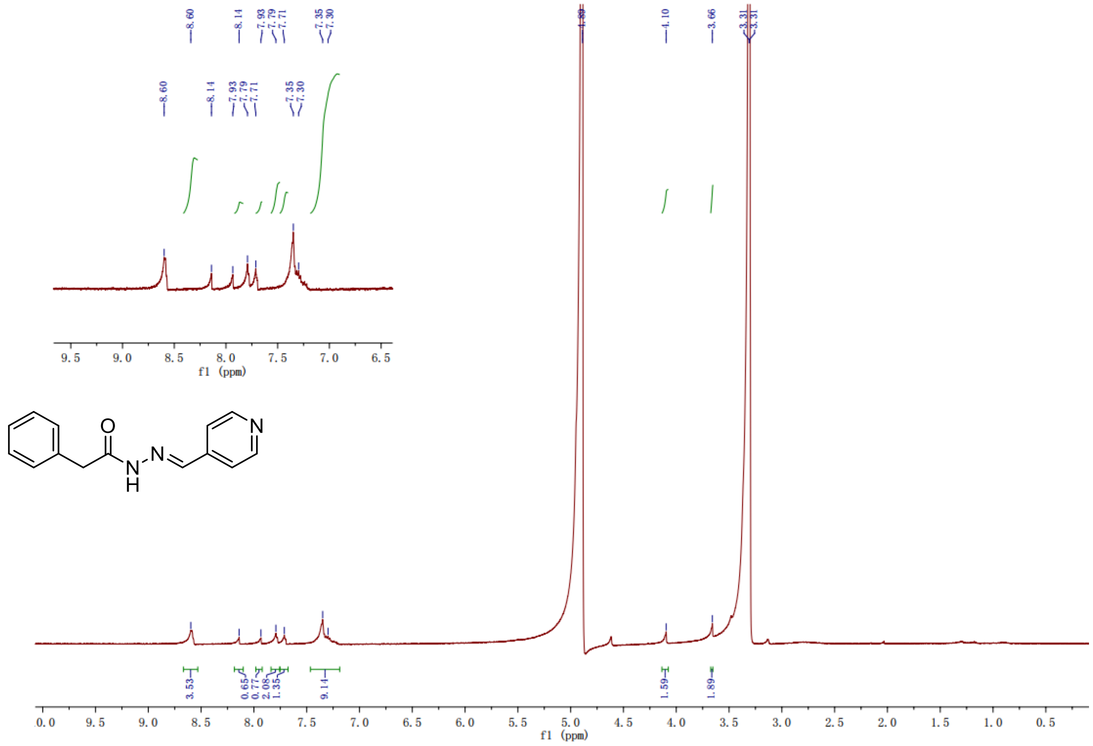

Figure S50. ^{1}H NMR (400 MHz) spectrum of 1 mM hydrazone compound 8 in MeOD-d_4.

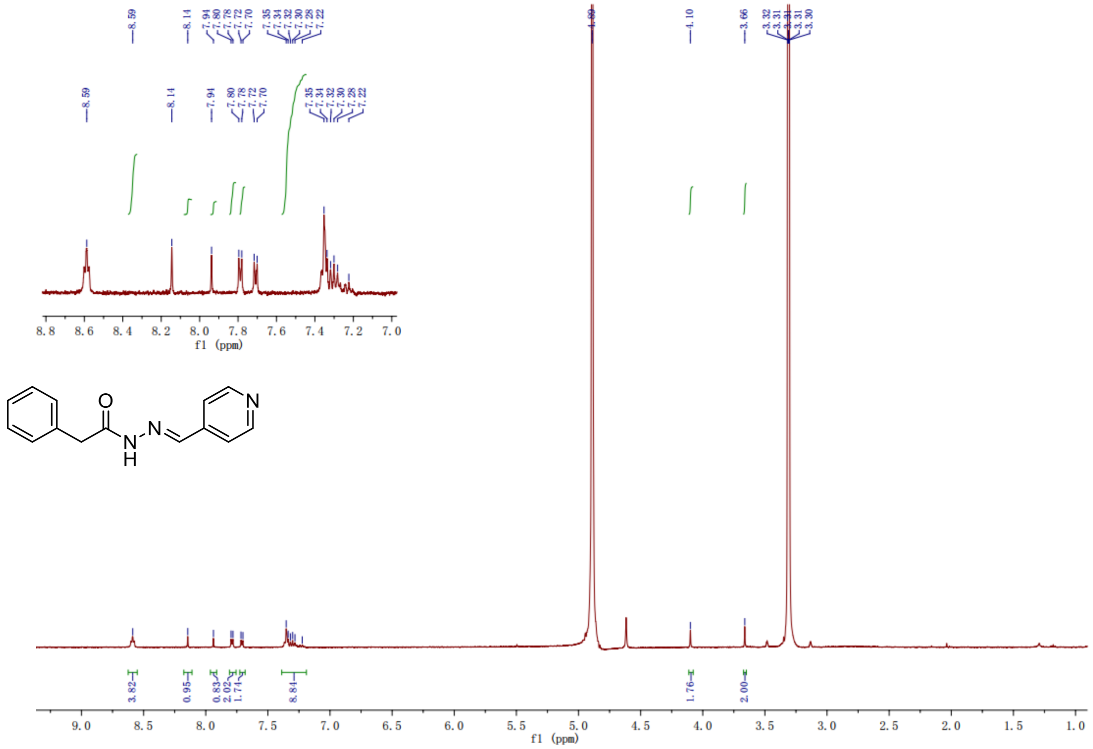

Figure S51. ^{1}H NMR (400 MHz) spectrum of 1 mM hydrazone compound 8 in MeOD-d_4 after bubbled CO and incubation.

Table S2. Palladium based CO probes.

#	Structure	FL profile	CO source used	Publication year	Ref
1	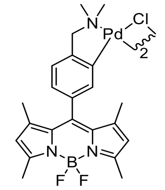 COP-1	λ_{ex}: 475 nm, λ_{em}: 503 nm	CORM-3	2012	8
2	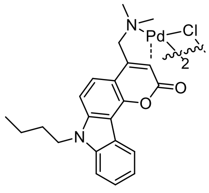 CC-CO	λ_{ex}: 350 nm λ_{em}: 365 nm	CORM-2	2014	9
3	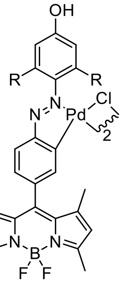 ACP-1: R=H ACP-2: R=-CH_3	λ_{ex}: 498 nm λ_{em}: 500-540 nm	CORM-2, CO gas	2016	10
4	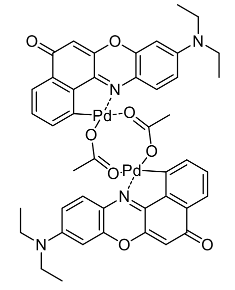 1-AC	λ_{ex}: 580 nm λ_{em}: 660 nm	CORM-2	2017	11
5	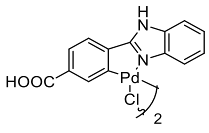 HFCO-1	λ_{ex}: 320 nm, λ_{em}: 415 nm,	CORM-2	2018	12
6	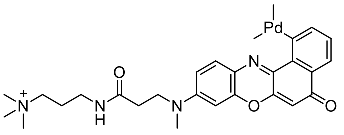 ANRP	λ_{ex}: 543 nm, λ_{em}: 650 nm,	CORM-2	2019	13
7	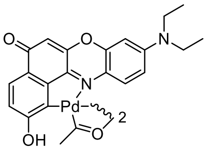	λ_{ex}: 530 nm, λ_{em}: 645 nm	Photo CORM and CO gas	2020	14

	Nile Red CO probe				
8	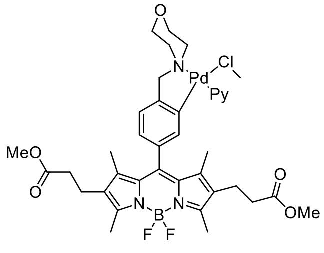 COP-3E-Py	λ_{ex}: 521 nm, λ_{em}: 535 nm	CORM-3	2020	[15]
9	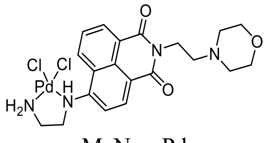 MeNap-Pd	λ_{ex}: 435 nm, λ_{em}: 532 nm	CORM-2	2021	[16]
10	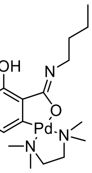 CODP-102	λ_{ex}: 397 nm, λ_{em}: 511 nm	CO gas, CORM, CO prodrug	2023	[17]
11	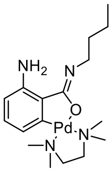 CODP-103	λ_{ex}: 386 nm, λ_{em}: 500 nm	CO gas, CORM, CO prodrug	2023	[17]
12	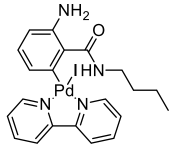 CODP-106	λ_{ex}: 386 nm, λ_{em}: 500 nm	CO gas, CORM, CO prodrug	2023	[17]
13	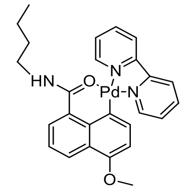 CODP-202	λ_{ex}: 377 nm, λ_{em}: 459 nm	CO gas, CORM, CO prodrug	2023	[17]

Table S3. CO probes based on de-allylation mechanism.

#	Structure	FL profile	CO source(s) used	Publication Year	Ref
1	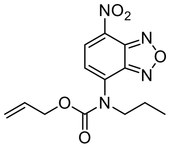 NBD-APC	λ_{ex}: 370 nm, λ_{em}: 549 nm	CORM-3 CO gas	2016	[18]

2	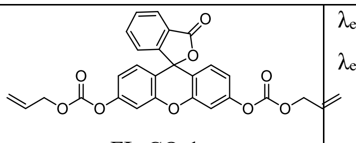 FL-CO-1	λ_{ex}: 490 nm, λ_{em}: 520 nm	CORM-3 CO solution	2016	[19]
3	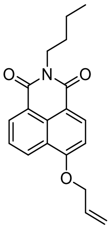 Ratio-CO	λ_{ex}: 430 nm, λ_{em}: 545 nm	CORM-2	2018	[20]
4	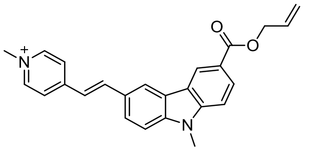 MPVC-II	λ_{ex}: 424 nm, λ_{em}:550 nm,	Bubbling CO	2018	[21]
5	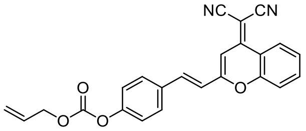 DCPO-probe 1	λ_{ex}: 565 nm, λ_{em}:685 nm,	CORM-3	2018	[22]
6	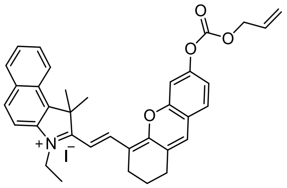 CyAPC	λ_{ex}: 690 nm, λ_{em}:736 nm,	CORM-3	2018	[23]
7	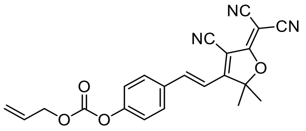 LW-CO	λ_{ex}: 550 nm, λ_{em}:605 nm,	CORM-2	2019	[24]
8	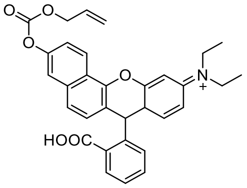 FR-CO	λ_{ex}: 450 nm, λ_{em}: 630 nm,	CORM-2	2019	[25]
9	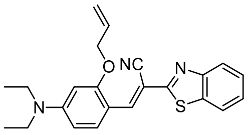	λ_{ex}: 465 nm, λ_{em}:546 nm (710 nm)	CORM-2	2019	[26]

	BTCV-CO				
10	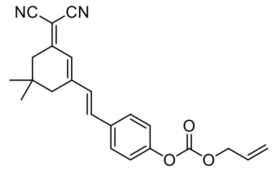<br>DCI-CO	λ_{ex}: 550 nm, λ_{em}: 700 nm	CORM-3	2019	[27]
11	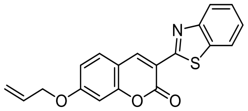<br>Cou-CO	λ_{ex}: 550 nm, λ_{em}: 700 nm	CORM-3	2019	[28]
12	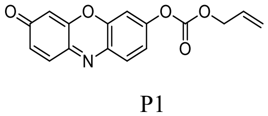<br>P1	λ_{ex}: 530 nm, λ_{em}: 585 nm	CO solution (1 mM)	2019	[29]
13	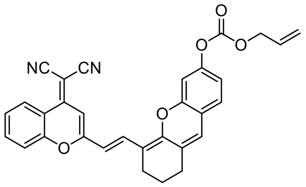<br>DCX-CO	λ_{ex}: 598 nm, λ_{em}: 762 nm	CORM-3	2020	[30]
14	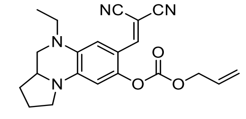<br>FP-1	λ_{ex}: 471 nm, λ_{em}: 608 nm	CORM-3	2021	[31]
15	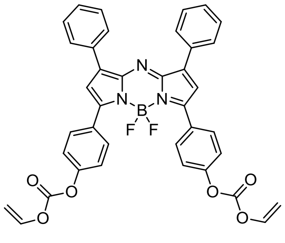<br>FP	λ_{ex}: 655 nm, 710 nm λ_{em}: 745 nm	CORM-3	2021	[32]
16	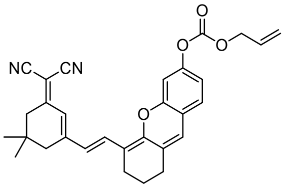<br>FDX-CO	λ_{ex}: 580 nm, λ_{em}: 685 nm	CORM-3	2021	[33]

17	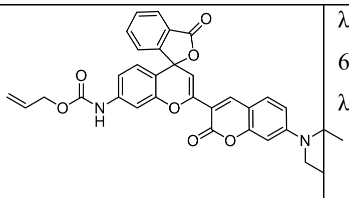 CP-CO	λ_{ex}: 420 nm, 610 nm, λ_{em}: 690 nm	CORM-3	2019	[34]
18	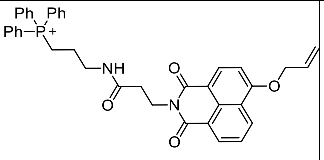 Mito-NIB-CO	λ_{ex}: 370 nm, 465 nm λ_{em}: 451 nm, 549	CORM-3	2021	[35]
19	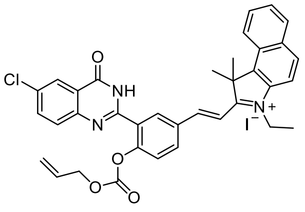 HPQ-BI-CO	λ_{ex}: 520 nm, 570 nm λ_{em}: 620 nm	CORM-3	2021	[36]
20	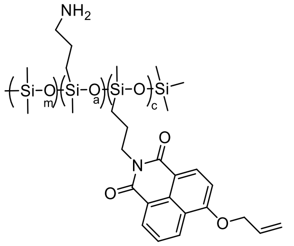 PMAH-CO	λ_{ex}: 425 nm, λ_{em}: 450nm, 559 nm	CORM-2	2021	[37]
21	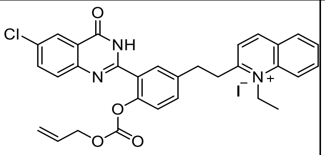 HPQ-MQ-CO	λ_{ex}: 550 nm, λ_{em}: 650 nm	CORM-3	2022	[38]
22	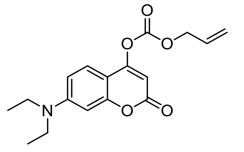 RTFP	λ_{ex}: 387 nm, λ_{em}: 470 nm	CORM-3	2023	[39]

Figures from the Original Publication

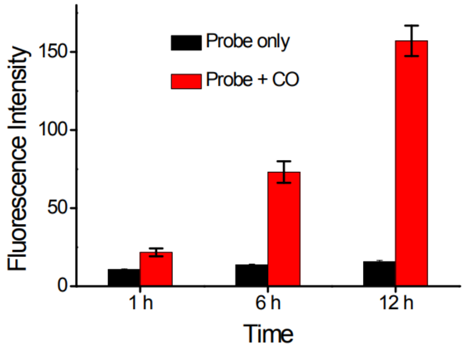

Figure S52. Original fluorescence intensity changes of RCO (10 µM) solution after continuous CO gas ventilation. $\lambda_{ex/em}$ = 525/578 nm. Figure taken from "*Chem. Commun.* **2019**, *55,* 9444" without modification.

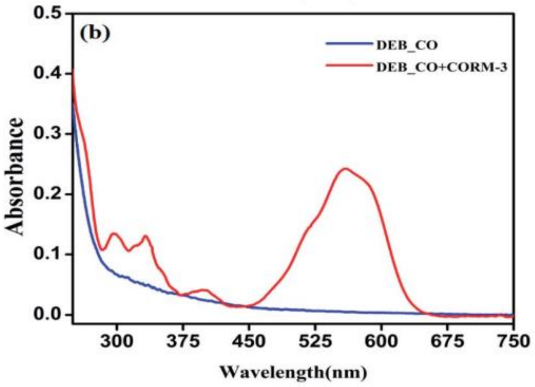

Figure S53. Original absorption spectra of 10 µM DEB-CO in pH 7.4 PBS before (blue) and after (red) reaction with 10 µM CORM-3 for 30 min. Figure taken from "*Anal Methods* **2022,** *14*, 3196" without modification.

Reference

1. C. Zhang, H. Xie, T. Zhan, J. Zhang, B. Chen, Z. Qian, G. Zhang, W. Zhang and J. Zhou, *Chem Commun*, 2019, **55**, 9444-9447.
2. E. Ahmmed, D. Sarkar, A. Mondal, N. C. Saha, S. Bhattacharyya and P. Chattopadhyay, *Anal Methods*, 2022, **14**, 3196-3202.
3. X.-F. Yang, X.-Q. Guo and Y.-B. Zhao, *Talanta*, 2002, **57**, 883-890.
4. G. Vantomme, S. Jiang and J.-M. Lehn, *J Am Chem Soc*, 2014, **136**, 9509-9518.
5. P. Nun, C. Martin, J. Martinez and F. Lamaty, *Tetrahedron*, 2011, **67**, 8187-8194.
6. A. Pal, K. M. Das, S. Sau and A. Thakur, *Chem Asian J*, 2023, **18**, e202300755.
7. H. S. Abdulrahman, M. Hassan Mohammed, L. A. Al-Ani, M. H. Ahmad, N. M. Hashim and W. A. Yehye, *J Chem*, 2020, **2020**, 3928204.
8. B. W. Michel, A. R. Lippert and C. J. Chang, *J Am Chem Soc*, 2012, **134**, 15668-15671.
9. K. Zheng, W. Lin, L. Tan, H. Chen and H. Cui, *Chem Sci*, 2014, **5**, 3439-3448.
10. Y. Li, X. Wang, J. Yang, X. Xie, M. Li, J. Niu, L. Tong and B. Tang, *Anal Chem*, 2016, **88**, 11154-11159.
11. K. Liu, X. Kong, Y. Ma and W. Lin, *Angew Chem Int Ed Engl*, 2017, **56**, 13489-13492.
12. M. Sun, H. Yu, K. Zhang, S. Wang, T. Hayat, A. Alsaedi and D. Huang, *ACS Sens*, 2018, **3**, 285-289.
13. S. Xu, H. W. Liu, X. Yin, L. Yuan, S. Y. Huan and X. B. Zhang, *Chem Sci*, 2019, **10**, 320-325.
14. D. Madea, M. Martinek, L. Muchova, J. Vana, L. Vitek and P. Klan, *J Org Chem*, 2020, **85**, 3473-3489.
15. J. Morstein, D. Hofler, K. Ueno, J. W. Jurss, R. R. Walvoord, K. J. Bruemmer, S. P. Rezgui, T. F. Brewer, M. Saitoe, B. W. Michel and C. J. Chang, *J Am Chem Soc*, 2020, **142**, 15917-15930.
16. S. Kumar Saha and N. Chandra Saha, *Inorganica Chimica Acta*, 2021, **517**, 120204.
17. X. Yang, Z. Yuan, W. Lu, C. Yang, M. Wang, R. Tripathi, Z. Fultz, C. Tan and B. Wang, *J Am Chem Soc*, 2023, **145**, 78-88.
18. Z. Y. Xu, J. W. Yan, J. Li, P. F. Yao, J. H. Tan and L. Zhang, *Tetrahedron Lett*, 2016, **57**, 2927-2930.
19. W. Y. Feng, D. D. Liu, S. M. Feng and G. Q. Feng, *Anal Chem*, 2016, **88**, 10648-10653.
20. Z. Wang, Z. Geng, Z. Zhao, W. Sheng, C. Liu, X. Lv, Q. He and B. Zhu, *New J Chem*, 2018, **42**, 14417-14423.
21. G. Shi, T. Yoon, S. Cha, S. Kim, M. Yousuf, N. Ahmed, D. Kim, H. W. Kang and K. S. Kim, *ACS Sens*, 2018, **3**, 1102-1108.
22. L. Yan, D. Nan, C. Lin, Y. Wan, Q. Pan and Z. Qi, *Spectrochim Acta A Mol Biomol Spectrosc*, 2018, **202**, 284-289.
23. S. J. Li, D. Y. Zhou, Y. F. Li, B. Yang, J. Ou-Yang, J. Jie, J. Liu and C. Y. Li, *Talanta*, 2018, **188**, 691-700.
24. Z. Wang, Z. Zhao, C. Liu, Z. Geng, Q. Duan, P. Jia, Z. Li, H. Zhu, B. Zhu and W. Sheng, *Photochem Photobiol Sci*, 2019, **18**, 1851-1857.
25. Z. K. Wang, Z. Y. Zhao, R. K. Wang, R. F. Yuan, C. Y. Liu, Q. X. Duan, W. W. Zhu, X. Y. Li and B. C. Zhu, *Anal. Methods*, 2019, **11**, 288-295.
26. J. Wang, C. Li, Q. Chen, H. Li, L. Zhou, X. Jiang, M. Shi, P. Zhang, G. Jiang and B. Z. Tang, *Anal Chem*, 2019, **91**, 9388-9392.
27. S. Gong, J. Hong, E. Zhou and G. Feng, *Talanta*, 2019, **201**, 40-45.
28. W. L. Fang, Y. J. Tang, X. F. Guo and H. Wang, *Talanta*, 2019, **205**.
29. B. Biswas, M. Venkateswarulu, S. Sinha, K. Girdhar, S. Ghosh, S. Chatterjee, P. Mondal and S. Ghosh, *ACS Appl Bio Mater*, 2019, **2**, 5427-5433.

30. Y. Liu, W. X. Wang, Y. Tian, M. Tan, Y. Du, J. Jie and C. Y. Li, *Dyes Pigments*, 2020, **180**.
31. J. Chen, Y. Gan, S. Hong, G. Yin, L. Zhou, C. Wang, Y. Fu, H. Li and P. Yin, *Anal Methods*, 2021, **13**, 2871-2877.
32. Z. Xu, A. Song, F. Wang and H. Chen, *RSC Adv*, 2021, **11**, 32203-32209.
33. Y. Tian, W. L. Jiang, W. X. Wang, J. Peng, X. M. Li, Y. Li and C. Y. Li, *Analyst*, 2021, **146**, 118-123.
34. W. L. Jiang, W. X. Wang, G. J. Mao, L. Yan, Y. Du, Y. Li and C. Y. Li, *Anal Chem*, 2021, **93**, 2510-2518.
35. F. Du, Y. Qu, M. Li and X. Tan, *Anal Bioanal Chem*, 2021, **413**, 1395-1403.
36. Y. S. Xia, L. Yan, G. J. Mao, W. L. Jiang, W. X. Wang, Y. F. Li, Y. Q. Jiang and C. Y. Li, *Sens Actuators B Chem*, 2021, **340**.
37. F. Gai, G. Ding, X. Wang and Y. Zuo, *Anal Chem*, 2021, **93**, 12899-12905.
38. G. Q. Fu, Y. S. Xia, W. L. Jiang, W. X. Wang, Z. K. Tan, K. Y. Guo, G. J. Mao and C. Y. Li, *Talanta*, 2022, **243**, 123398.
39. J. Tang, P. Zhang, Z. Li, Y. Zhang, H. Chen, X. Li and C. Wei, *Bioorg Chem*, 2023, **135**, 106489.
